# annotate_my_genomes: an easy-to-use pipeline to improve genome annotation and uncover neglected genes by hybrid RNA sequencing

**DOI:** 10.1093/gigascience/giac099

**Published:** 2022-12-06

**Authors:** Carlos Farkas, Antonia Recabal, Andy Mella, Daniel Candia-Herrera, Maryori González Olivero, Jody Jonathan Haigh, Estefanía Tarifeño-Saldivia, Teresa Caprile

**Affiliations:** Laboratorio de Investigación en Ciencias Biomédicas, Departamento de Ciencias Básicas y Morfología, Facultad de Medicina, Universidad Católica de la Santísima Concepción, Concepción, Chile; Departamento de Biología Celular, Facultad de Ciencias Biológicas, Universidad de Concepción, Chile; Instituto de Ciencias Naturales, Universidad de las Américas, Chile; Centro Integrativo de Biología y Química Aplicada (CIBQA), Universidad Bernardo O'Higgins, Santiago 8370854, Chile; Departamento de Bioquímica y Biología Molecular, Facultad de Ciencias Biológicas, Universidad de Concepción, Chile; Departamento de Biología Celular, Facultad de Ciencias Biológicas, Universidad de Concepción, Chile; CancerCare Manitoba Research Institute, Winnipeg, MB, Canada; Department of Pharmacology and Therapeutics, Rady Faculty of Health Sciences, University of Manitoba, Winnipeg, MB, Canada; Departamento de Bioquímica y Biología Molecular, Facultad de Ciencias Biológicas, Universidad de Concepción, Chile; Departamento de Biología Celular, Facultad de Ciencias Biológicas, Universidad de Concepción, Chile

**Keywords:** Transcriptome annotation, Genome Annotation pipeline, SCO-spondin, hybrid sequencing

## Abstract

**Background:**

The advancement of hybrid sequencing technologies is increasingly expanding genome assemblies that are often annotated using hybrid sequencing transcriptomics, leading to improved genome characterization and the identification of novel genes and isoforms in a wide variety of organisms.

**Results:**

We developed an easy-to-use genome-guided transcriptome annotation pipeline that uses assembled transcripts from hybrid sequencing data as input and distinguishes between coding and long non-coding RNAs by integration of several bioinformatic approaches, including gene reconciliation with previous annotations in GTF format. We demonstrated the efficiency of this approach by correctly assembling and annotating all exons from the chicken SCO-spondin gene (containing more than 105 exons), including the identification of missing genes in the chicken reference annotations by homology assignments.

**Conclusions:**

Our method helps to improve the current transcriptome annotation of the chicken brain. Our pipeline, implemented on Anaconda/Nextflow and Docker is an easy-to-use package that can be applied to a broad range of species, tissues, and research areas helping to improve and reconcile current annotations. The code and datasets are publicly available at https://github.com/cfarkas/annotate_my_genomes

## Background

The emergent advancement of Next Generation Sequencing (NGS) combined with novel genome assembly methods greatly improved genome characterization, identifying novel genes and isoforms in both model as well as non-model organisms [1–3]. RNA-sequencing (RNA-seq) based on short reads resolve transcriptomes in a limited manner due to technical limitations in assembly [4]. Long-read RNA-seq technologies alone or combined with short-read sequencing often improve the quality and contiguity of transcriptome assemblies [5, 6]. Long-read technologies such as PacBio single-molecule real-time (SMRT) and Oxford Nanopore (ONT) sequencing technologies (hereafter PacBio and Nanopore sequencing, respectively) are more efficient than short-read RNA-seq to reconstruct full-length transcripts by using error correction and polishing pipelines [7]. Well-established PacBio-only based pipelines such as IsoSeq [8, 9] and IsoCon [10] often perform well on these tasks and hybrid sequencing even outperforms these methods producing better transcriptome assemblies [11–13]. After assessing the best transcriptome assembly with tools such as rnaQUAST [14], SQANTI [15], or by using multiple assemblies to improve gene structure annotation [16], an additional challenge in transcriptomic studies is the feature identification and annotation process. Initially, pipelines such as MAKER integrated trained *ab initio* gene predictions, Expressed Sequence Tags (EST), and proteins to annotate genes from a given genome [17, 18]. In the same way, the gene prediction program AUGUSTUS accurately predicts genes using supervised training of EST and proteins as external hints, including the use of short read RNA-seq alignments to improve final gene prediction [19, 20]. Later, BRAKER1 pipeline was developed, a short read RNA-seq genome annotation pipeline that combines AUGUSTUS and GeneMark-ET, an unsupervised RNA-seq gene prediction tool [21, 22]. Subsequently, BRAKER2 improved BRAKER1 work by integrating iterative-training gene predictions from GeneMark-ET and AUGUSTUS, transcriptomic data, and external protein support altogether [23]. More recently, the TSEBRA pipeline selects transcripts from BRAKER1 and BRAKER2 predictions altogether, by ranking all transcript predictions according to the RNA-seq and homologous protein evidence support and selecting the best candidates [24]. Evidence-based proteomics and transcriptomics for gene-finding, offers complete and reliable genome annotations, but dedicated tools for hybrid RNA-seq analysis are also needed. Regarding the latter, the long-read annotation tool LoReAN combines Trinity-based transcript assemblies and BRAKER1 predictions from short-read RNA-seq, including clustered transcript reconstruction from long-read sequencing technologies and proteome data as well [25]. Although more effective than short-read annotation pipelines, the latter pipeline can be time and CPU consuming, especially in the use of Trinity assembly process when large datasets are employed. In the present work, we present annotate_my_genomes, an easy-to-use transcriptome annotation pipeline that uses assembled transcripts from hybrid sequencing data as input and distinguishes between coding and long non-coding RNAs (lncRNAs) by integration of several well-established approaches, including gene reconciliation with previous annotations. This method requires a reference genome as a guide and leads to superior transcriptome assembly and annotation when compared to traditional Illumina or PacBio RNA-seq protocols such as IsoSeq as well as similar pipelines [26, 27]. We demonstrated the efficiency of this approach by correctly assembling all exons from the chicken SSPO gene (containing more than 105 exons) and mapping missing genes in the chicken reference genome by homology assignments. We demonstrated that using StringTie GTF assembly as input, our method tends to improve the current genome annotation, surpassing BRAKER1/2 and TSEBRA performances. Importantly, the presented data provides the first transcriptional landscapes of sub-commissural organ (SCO) of the chick embryo, a brain gland related to different morphogenic events, such as the regulation of brain development and body axis alignment [28, 29].

## Data Description

The transcriptome of the chick embryo sub-commissural organ (SCO) was performed using a combination of Illumina (short-reads) and PacBio (long-reads) sequencing. To prepare the samples, we dissected and pooled 25 SCOs from outbred Gallus gallus embryos at Hamburger-Hamilton (HH) stages HH23 and HH30. Total RNA was isolated using the RNeasy Mini Kit (QIAGEN). The concentration and quality of RNA were measured using Qubit™ RNA HS Assay Kit (RIN values between 8.8–9.5 per sample). Four PacBio RSII Isoform libraries were constructed by using 2 µg of total RNA from HH23 (n = 2) and HH30 (n = 2) SCO (Cold Spring Harbor Laboratory, Genomic Platform, USA). Sequencing was performed by using IsoSeq protocol (Pacific Biosciences) with long (>4 kb) and standard library enrichment sizes per stage. TruSeq Illumina libraries were prepared (two replicates by sample) and sequenced on a NextSeq Paired-End 150 bp middle output (Cold Spring Harbor Laboratory, Genomic Platform, USA). PacBio and Illumina RNA-sequencing datasets are available at European Nucleotide Archive (ENA) Accession Number PRJEB36569 (PacBio) and PRJEB36584 (Illumina).

## Analyses

### Combined PacBio and Illumina RNA sequencing assembly improved gene annotation in the chicken transcriptome

**Figure 1:**
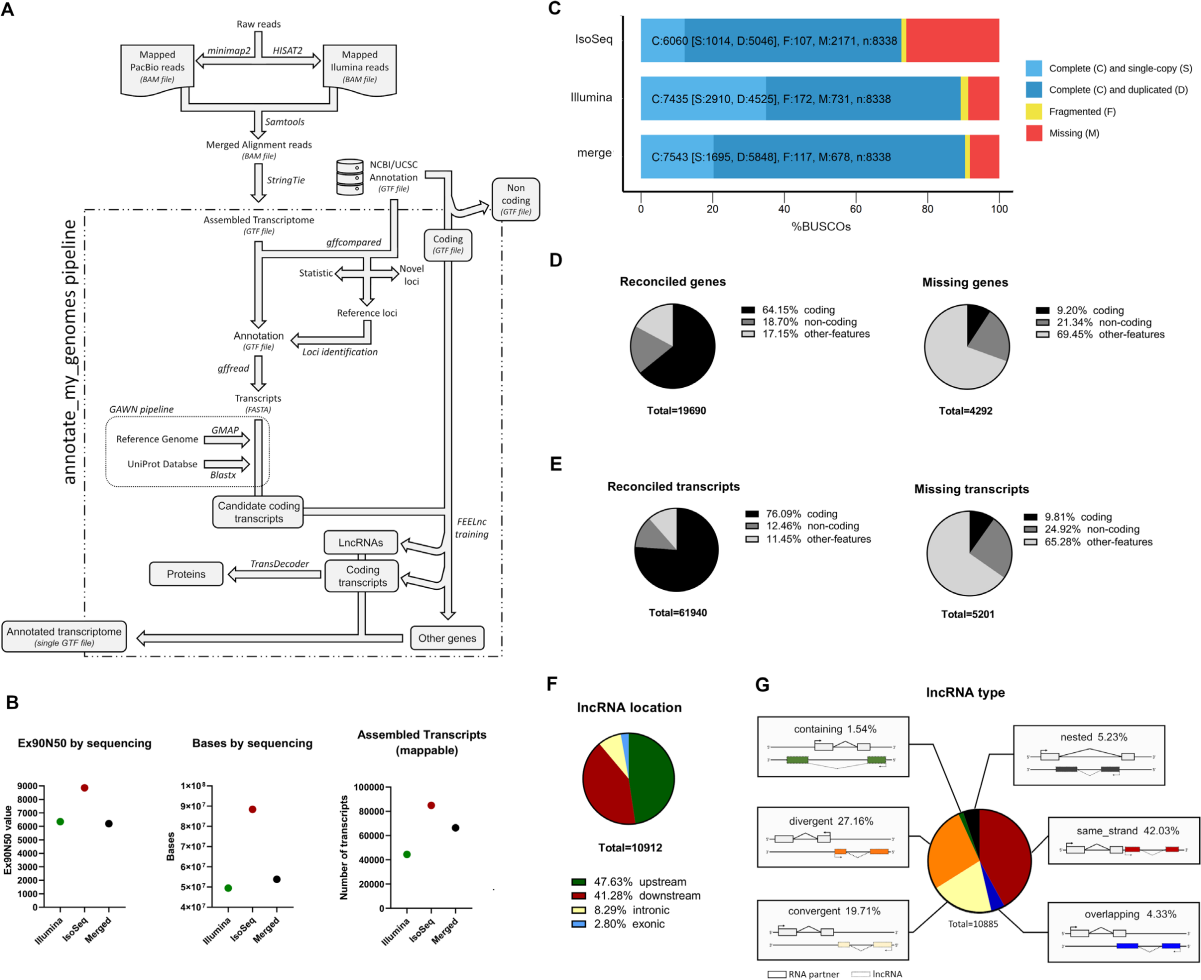
Combined PacBio and Illumina RNA sequencing assembly improves gene annotation in the chicken transcriptome. **A**) Schematic diagram of annotate_my_genomes pipeline. PacBio and Illumina reads are aligned to a given reference genome, then the alignments were merged and assembled into isoforms by *StringTie*. By combined homology identification and coding/noncoding RNA classification, we annotated the resulting GTF file, identifying previously annotated genes (i.e., USCS/NCBI) and missing genes, respectively (see annotate_my_genomes). **B**) Various transcriptome metrics for StringTie assemblies based solely on Illumina, PacBio (IsoSeq), and merge of the alignments from both technologies (see green, red, and black dots, respectively). From left to right, we show Ex90N50, bases, and number of assembled transcripts, respectively. **C**) BUSCO classification of single-copy and multicopy orthologs in each transcriptome assembly. Complete, fragmented, and missing orthologs are depicted with different colors. **D**) (*left*) Number of coding, noncoding, and other assembled transcripts (other features) in reconciled 19690 genes with NCBI current annotation for galGal6 in June 2020. (*Right*) Same as left for 4292 non annotated genes in NCBI current annotation for galGal6 in June 2020. **E**) Same as (D) for the number of transcripts. **F**) Classification of 10912 annotated long noncoding RNAs by location using FEELnc tool. **G**) Classification of lncRNA by Type using FEELnc tool.

To uncover the transcriptome of chicken SCO organs at Hamburger-Hamilton (HH) stages HH23 and/or HH30 stages, we performed a hybrid sequencing approach using long (>4 kb) and standard library enrichment sizes for PacBio, and Illumina platforms. Instead of assembling PacBio reads by traditional pipelines such as IsoSeq [26, 27] and/or IsoCon [10], we aligned reads against the Genome Reference Consortium Chicken Build 6a (GRCg6a) reference genome (assembly GCF_000002315.5) by using *minimap2*, a splice aware aligner [30]. Illumina short reads were trimmed using fastp tool [31] and aligned using HISAT2 [32]. Posteriorly, the alignments from both technologies were merged. Transcripts were assembled from merged aligned reads using *StringTie* program and transcripts were annotated based on the NCBI/UCSC annotation associated with GRCg6a assembly (March 2018 version). Assembled transcripts in GTF format were used as input for our annotate_my_genomes pipeline. First, assembled transcripts were identified based on homology with BLASTX [33]. New transcripts (not included on the genome annotation) were further classified as coding or non-coding, using the long non-coding RNA classification tool *FEELnc* [34]. Non-lncRNA transcripts presenting a BLASTX match were further collected, and open reading frames were predicted by using *TransDecoder* gene coding prediction pipeline (https://github.com/TransDecoder/TransDecoder). Finally, we employed the UniProt database [35] to identify novel coding transcripts (Fig. 1A). With this setting, we benchmarked the quality of these assemblies produced by each technology including the merged alignment approach. Ex90N50 values from IsoSeq transcriptome assembly surpassed both the merged alignment and Illumina-alone transcriptome assemblies (Fig. 1B, left). The merged alignment assembly improved Illumina bases per sequencing and mappable assembled transcripts, suggesting an overall improvement of Illumina-alone sequencing technology, while IsoSeq assembly surpassed both merged alignment assembly and Illumina-alone assemblies (Fig. 1B, middle and right, respectively).

Even though IsoSeq displayed better assembly statistics than Illumina technology, Illumina and merged alignment of both sequencing technologies led to higher-quality assemblies assessed by the number of completed BUSCOs found in the *aves* lineage (aves_odb10) (Fig. 1C). This result is expected since Illumina technology has better sequencing depth and quality than IsoSeq (∼1% versus ∼11% overall sequencing error, respectively) [36]. Also, the merged strategy slightly improved the completeness of the SCO transcriptome compared to the Illumina-alone assembly (1%). Thus, despite higher Ex90N50 values of IsoSeq assembly, we further selected the merged alignment assembly strategy to annotate genes, because of its increased quality over Illumina and PacBio-alone assemblies.

Our pipeline identified 19690 reconciled genes including 4292 candidate genes that are not annotated on the chicken GRCg6a reference genome, from them 64% and 9% corresponding to coding genes (Fig. 1D, left and right, respectively). At the level of transcripts, we observed 61940 reconciled and 5201 non-annotated transcripts, where 76% and ∼10% are coding transcripts, respectively (see Fig. 1E, left and right, respectively, and Supplementary Table 1). Of note, a substantial number of transcripts not classified as either coding or long non-coding RNAs composed missing transcripts (65%), arguing that these transcripts survived the RNA quality control system removal from the cell [37, 38] and could correspond to small RNAs, incomplete gene models, and/or transcripts emanating from repeat regions [39]. We aimed to classify the discovered long noncoding RNAs (lncRNAs) by location and subtype, by using the FEELnc classification tool [34]. By location, concerning neighboring genes, we found a significant proportion of exonic/intronic lncRNAs types (∼11%, Fig. 1F). Also, by orientation, a significant proportion of all lncRNAs are divergent lncRNAs (27%, Fig. 1G). Overall, our results confirm that hybrid sequencing is beneficial for a comprehensive and reconciliated characterization of a given transcriptome, which agrees with a previous report [25] and a more recent report [40]. In addition, our tool provides a way to streamline the annotation process in a user-friendly manner.

### The Giant SSPO gene is fully reconstructed by hybrid sequencing technology.

**Figure 2:**
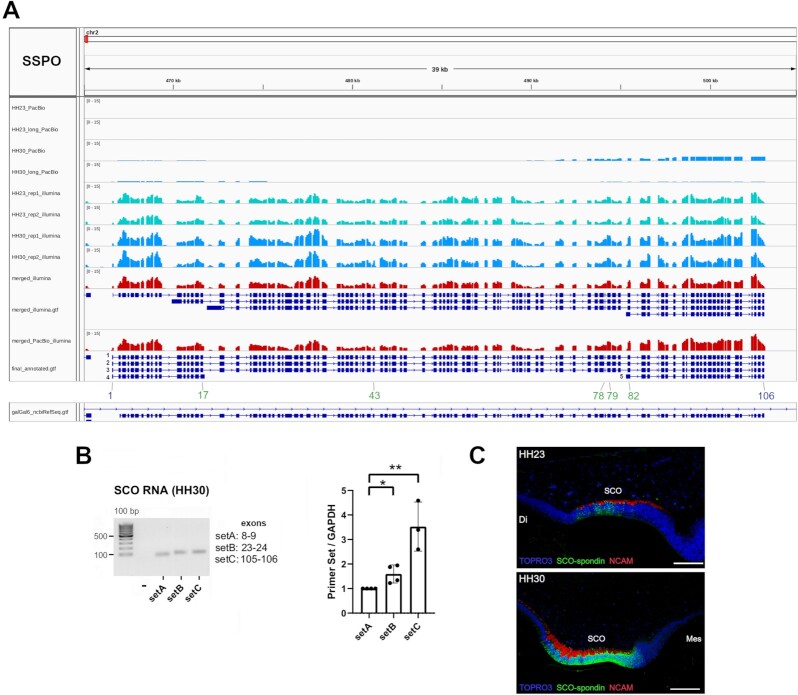
Giant SSPO gene is fully reconstructed by hybrid sequencing technology. **A**) Coverage of PacBio and Illumina alignment at *SSPO* locus (chr2:466581–503024 in galGal6 assembly) at HH23 (magenta) and HH30 (blue) stages, visualized by IGV viewer. The red-colored track indicates the coverage of merged Illumina-only BAM file including the correspondent assembled isoforms underneath in blue. Similarly, we included as a red-colored track the coverage of merged PacBio and Illumina sequencing alignmemts including the assembled isoforms underneath in blue. We highlighted in blue numbers the beginning and last exons of assembled *SSPO* isoforms and in green numbers the alternative isoform usage across *SSPO* isoforms. We included the current galGal6a annotation underneath all tracks in blue color. **B**) (*left*) PCR product of three sets of primers spanning SSPO gene at exons 8–9 (setA), 23–24 (setB) and 105–106 (setC), from RNA of SCO at stage HH30. (*right*) qPCR of the three referred primer sets from SCO RNA derived from at least ten pooled animals coming from four different egg laying at stage HH30. Significance of comparisons was assessed with Student's t test (*P*<0.05 *, *P*<0.01 **, *P*<0.001 ***, *P*>0.05 ns) **C**) Immunohistochemistry of SCO-spondin and NCAM in the SCO at HH23 (upper) and HH30 (lower) stages. TOPRO3 in blue, NCAM in red, and SCO-spondin in green, Di: Diencephalon, Mes: Mesencephalon. Significance of comparisons were assessed with Student's t test (*P*<0.05 *, *P*<0.01 **, *P*<0.001 ***, *P*>0.05 ns).

We aimed to assemble and annotate with our pipeline the giant gene SSPO, a 105-exon gene encoding the main secreted glycoprotein that forms the Reissner fiber from the subcommissural organ [41, 42]. The SSPO gene in chicken is provisionally classified as a protein coding gene by NCBI (https://www.ncbi.nlm.nih.gov/gene/420367), thus, we challenged our hybrid alignment assembly coupled with our annotation pipeline with this giant gene. Previous whole-brain Illumina sequencing at HH31/HH36 stages did not contain any mapped read to SSPO locus, probably because SCO is the unique source of SSPO expression (Supplementary Fig. 1). The latter also explains the consistent absence of SSPO in coding reference annotations due to lack of SCO transcriptome data (see https://www.ensembl.org/Gallus_gallus_GCA_000002315.5/Gene/Summary?db=core;g=ENSGALG00000033417;r=2:466594-502832;t=ENSGALT00000058788). We examined the coverage of PacBio, Illumina, and the merge of HH23 and HH30 RNA sequencing, demonstrating that PacBio sequencing fails to properly assemble the SSPO gene (SCO-spondin), one of the main secreted glycoproteins from the SCO (Fig. 2A). Also, SSPO related transcripts did not figure in the circular consensus sequences (CCS) or in the high- or low-quality assembled transcripts from IsoSeq (https://github.com/ben-lerch/IsoSeq-3.0). Conversely, Illumina sequencing from HH23/HH30 stages aligned to SSPO locus (see turquoise and blue colored tracks in Fig. 2A, respectively). Combined Illumina-PacBio sequencing led to the assembly of four SSPO isoforms of 106, 95, 89, and 25 exons, respectively (see the red-colored track and gene track underneath in Fig. 2A). The merged strategy led to the assembly of two SSPO isoforms of 106 exons, and 3 isoforms of 105, 17, and 25 exons, all of them classified to encode proteins and not lncRNAs (see transcripts 1, 3, 2, 4, and 5 in Gene_PacBio_Illumina track from Fig. 2A, respectively). Transcript 1 encodes a protein of 5270 amino acids with 98.88% of identity with a previously deduced SSPO protein in chicken, derived from a cloned cDNA in SCO (GenBank accession AJ 866919) [43]. The latter protein contains 5255 amino acids encoded within 105 exons, lacking the first assembled exon of our reconstructed transcripts. Thus, the merged alignment strategy leads to a superior assembly consisting of five alternative isoforms (see green-colored numbers indicating the new exons in Fig. 2A). Also, the use of Illumina-only assembly leads to an incomplete assembly of SSPO gene at the 5′ end (see Illumina gene track in Fig. 2A, the track called “merged_illumina.gtf”). Regarding the latter, no degradation of 5′ or 3′ ends of SSPO gene transcripts was detected after coverage inspection of SCO Illumina datasets with the RSeQC package [44] (Supplementary Fig. 2). By using sets of primers designed at the beginning and the end of SSPO gene, we confirmed increased transcription at the 3′ end of SSPO at stage HH30 in the SCO, supporting the existence of a C-terminal isoform (Fig. 2B). Immunohistochemistry of SCO at stages HH23 and HH30 confirm increased expression of SCO at HH30 (Fig. 2C), as previously confirmed by western blot [28]. In summary, hybrid sequencing leads to better assemblies by improving contiguity and lowering misassembles with the combination of PacBio long reads and high-quality Illumina short reads, respectively.

## Pipeline Benchmarking

**Figure 3:**
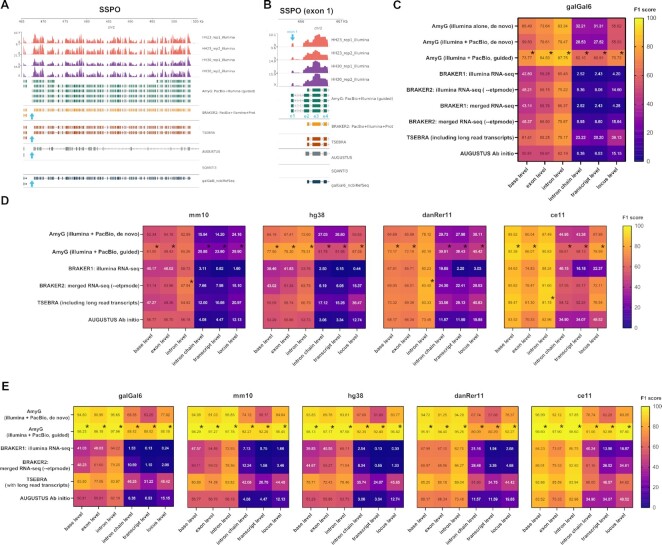
Pipeline benchmarking. A) Coverage of PacBio and Illumina alignments at SSPO locus (chr2:466581–503024 in galGal6 assembly) at HH23 (red) and HH30 (purple) stages, plotted with pyGenomeTracks, including SSPO gene models resolved with annotate_my_genomes (input: genome-guided StringTie assemblies from PacBio+Illumina merged alignments, green tracks), BRAKER2 (yellow tracks), TSEBRA (brown tracks), AUGUSTUS (grey tracks), SQANTI3 (no tracks to plot at this position) and galGal6_ncbiRefSeq (dark blue track), respectively. The latter track corresponds to the reference track provided by NCBI. The light blue-colored track indicates a novel assembled exon corresponding to the first exon of SSPO (exon 1). **B)** Detailed view of the first assembled exon by annotate_my_genomes, Light-blue annotations indicate exon numbers. **C)** Heatmap depicting F1-score calculations of each hybrid RNA-seq assembly method on Gallus gallus. We compared the output GTF from each method against the NCBI annotations (galGal6_ncbiRefSeq.gtf) on the base, exon, intron, intron-chain, transcript, and locus level parameters, respectively. All employed methods are enlisted on the left of the graph. The F1 score was calculated based on precision and recall values obtained on each parameter from gffcompare. Black asterisks indicate the best F1 scores per method. The color scale indicates lower and higher F1 values with blue and yellow scales, respectively. AmyG = annotate_my_genomes. **D)** Same as C) for real Mus musculus (mm10), Homo sapiens (hg38), Danio rerio (danRer11) and Caenorhabditis elegans (ce11) hybrid RNA-seq datasets, respectively. The benchmarked methods were the following: annotate_my_genomes (with or without genome guide) BRAKER1, BRAKER2, TSEBRA and AUGUSTUS *Ab initio*. Black asterisks indicate the best F1 scores per method. The color scale indicates lower and higher F1 values with blue and yellow scales, respectively. AmyG = annotate_my_genomes. **E)** Same as D) for simulated Gallus gallus, Mus musculus (mm10), Homo sapiens (hg38), Danio rerio (danRer11) and Caenorhabditis elegans (ce11) datasets. AmyG = annotate_my_genomes.

We initially inspected the assembly of SSPO gene in *Gallus gallus* genome (galGal6) from annotate_my_genomes pipeline including dedicated genome annotation pipelines such as SQANTI3 [15], BRAKER1 [21], BRAKER2 [23], including the recent TSEBRA method [24]. AUGUSTUS *ab initio* predictions were also included in the comparison [20]. BRAKER2, TSEBRA and annotate_my_genomes, but not *ab initio* AUGUSTUS and SQANTI3 methods correctly assembled all previously described exons from SSPO (n = 105) (Fig. 3A). Of notice, annotate_my_genomes method assembled these 105 exons, including an additional exon (hereafter exon 1) across SSPO isoforms (Fig. 3B). These preliminary results indicate that our method can resolve more exons than the referred methods, however this result might not necessarily imply a better assembly.

Therefore, we additionally annotated *Mus musculus* (mm10), *Homo sapiens* (hg38), *Danio rerio* (danRer11) real RNA-seq datasets sequenced with short and long reads, using the referred annotation methods (see Supplementary Table 4 for sequencing datasets). We also included in the analysis a nanopore-only direct RNA sequencing of *Caenorhabditis elegans* (ce11) embryos, consisting in long reads spanning the full length of mRNA transcripts [45]. We compared the gene annotation predictions from each method in GTF format against the NCBI reference GTF from each genome (the latter considered as truth), using gffcompare [46]. The evaluated parameters covered bases, exon, intron, intron-chain, transcript, and locus level assessments, as described here: https://ccb.jhu.edu/software/stringtie/gffcompare.shtml). For each evaluated parameter, gffcompare retrieved precision ($\frac{{TN}}{{FP + TN}}$, where TN = True Negatives and FP = False Positives, respectively) and recall ($\frac{{TP}}{{FN + TP}}$, where TP = True Positives and FN = False Negatives). Then, we calculated the harmonic mean of the latter values (F1-score) as follows: $\frac{{2x( {precision\ x\ recall} )}}{{precision + recall}}$. We considered F1-score as the final measure of gene prediction accuracy for each method. In all evaluated parameters, annotate_my_genomes coupled with a genome-guided StringTie GTF assembly derived from short and long read alignments, outperform all methods in our sequencing dataset (galGal6) (Fig. 3C, see asterisks). This behaviour was also seen in *Mus musculus, Homo sapiens, Danio rerio* and *Caenorhabditis elegans*, excepting at the intron level, where BRAKER2 and/or TSEBRA methods outperform our method in three out of four datasets (Fig. 3D, see asterisks). annotate_my_genomes coupled with a *de-novo* StringTie GTF assembly derived from short and long read alignments performed similarly as BRAKER2 or TSEBRA in each dataset, sometimes surpassing BRAKER2/TSEBRA (see galGal6 in Fig. 3C and mm10, hg38 in Fig. 3D, respectively).

We also noticed CPU times were equal or inferior when annotate_my_genomes method is employed, in comparison with BRAKER1, BRAKER2, or TSEBRA across the referred RNA-seq datasets (Supplementary Fig. 3).

To overcome coverage and noise variability from real datasets, we simulated PacBio, and Illumina reads using IsoSeqSim tool (https://github.com/yunhaowang/IsoSeqSim) and ReSeq tool, respectively, as described in Material and Methods. As ReSeq need real Illumina datasets as input, we included in the simulations the referred Illumina datasets for each species including an additional illumina dataset of *Caenorhabditis elegans* L1 larvae cells (PRJNA733501) to enable fair comparisons across all species for hybrid datasets. Simulated PacBio and Illumina datasets were then aligned against their reference transcriptomes using *minimap2* and *HISAT2* aligners, respectively and the resulting simulated and mapped transcriptomes were merged and assembled using *StringTie*. The resulting GTF files from the assemblies were inputted into annotate_my_genomes pipeline while the individual Illumina and PacBio alignments in BAM format were used as inputs for BRAKER1/2 and TSEBRA pipelines, respectively.

Similarly, as observed with the real datasets benchmarking, annotate_my_genomes coupled with a *de-novo* and/or genome-guided StringTie GTF assembly derived from short and long read alignments, outperformed all referred methods in the simulations across all evaluated parameters (Fig. 3E). In particular, our pipeline in combination with genome-guided StringTie assemblies outperforms every single method. The latter confirms the trends observed in the benchmarking employing real datasets.

**Figure 4:**
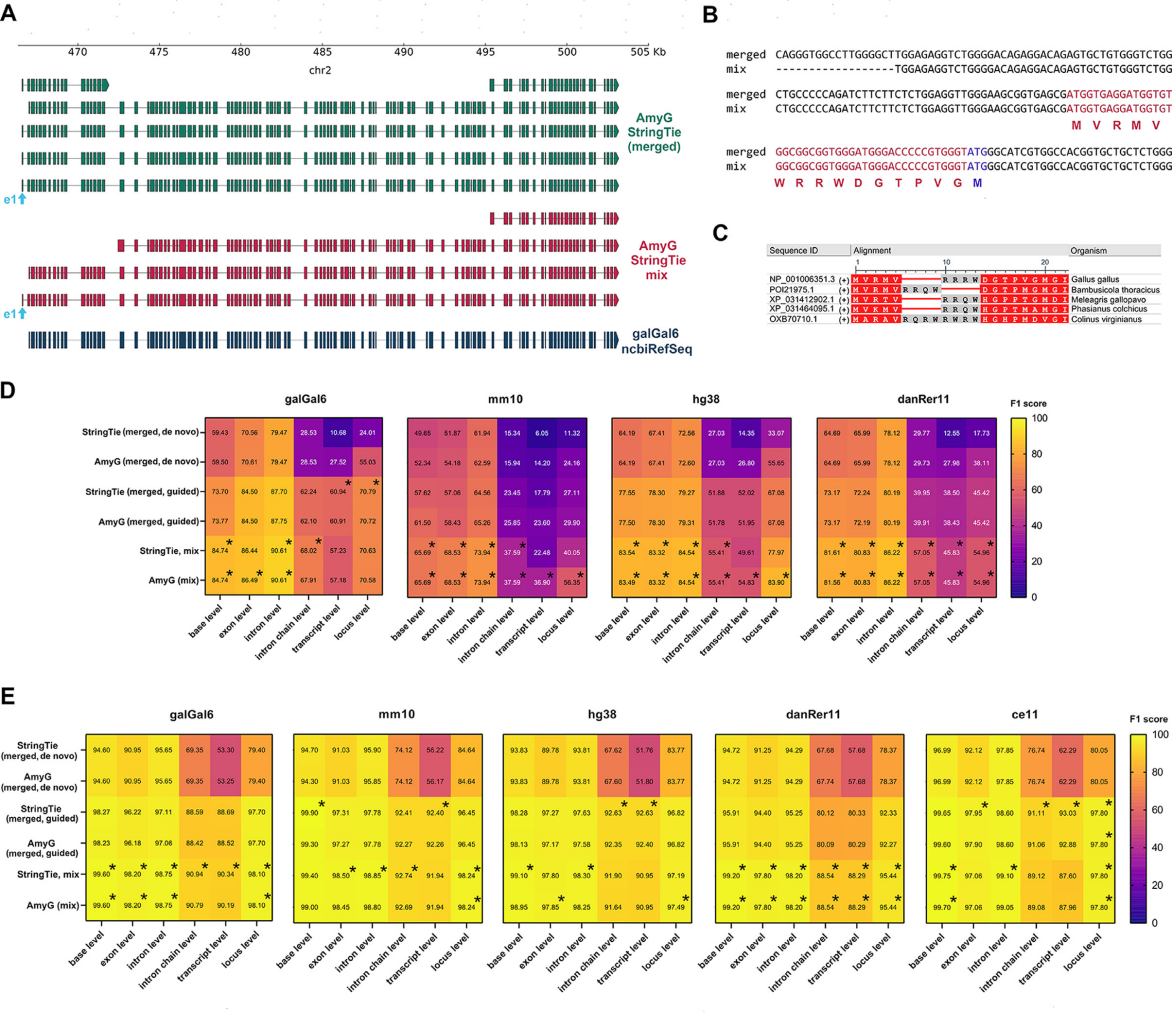
Benchmarking on StringTie-mix method. **A)** SSPO gene models resolved with annotate_my_genomes + genome-guided StringTie assembly derived from PacBio+Illumina merged alignment data (green tracks), annotate_my_genomes + genome-guided StringTie-mix assembly (red tracks), and galGal6_ncbiRefSeq (dark blue track), respectively. The latter track corresponds to the reference track provided by NCBI. The light blue-colored track indicates a novel assembled exon corresponding to the first exon of SSPO (exon 1). **B)** Genomic sequence of exon 1 and 2 from assembled SSPO transcript models from the latter approaches (merged = PacBio+Illumina genome-guided StringTie assembly, mix = PacBio+Illumina genome-guided StringTie mix assembly). Aminoacid sequences and correspondent complementary DNA are highlighted in red. Previously characterized translation start site is highlighted in blue. **C)** Protein BLAST (blastp) + COBALT analysis identified four transcripts from different *Aves* class members harboring N-terminal peptide assembled by our pipeline. Homology confident aminoacids are highlighted in red. **D)** Heatmap depicting F1-score calculations of raw StringTie GTF annotations derived from merged PacBio and Illumina alignments assemblies, using *de-novo* assembly, genome-guided assembly and genome-guided StringTie-mix assemblies, respectively. Each StringTie raw annotation was processed with annotate_my_genomes pipeline, respectively (AmyG = annotate_my_genomes). These methods were benchmarked on real Gallus gallus (galGal6), Mus musculus (mm10), Homo sapiens (hg38) and Danio rerio (danRer11) datasets. We compared the output GTF from each method against the reference NCBI annotations on the base, exon, intron, intron-chain, transcript, and locus level parameters, respectively on every species. The F1 score was calculated based on precision and recall values obtained on each parameter from gffcompare. Black asterisks indicate the best F1 scores per method. The color scale indicates lower and higher F1 values with blue and yellow scales, respectively. **E)** Same as D) for Gallus gallus (galGal6), Mus musculus (mm10), Homo sapiens (hg38), Danio rerio (danRer11) and Caenorhabditis elegans (ce11) simulated datasets.

During the review of this manuscript, a novel version of *StringTie* was released with the aim to improve the identification of novel gene isoforms in hybrid sequencing datasets. The new method employs the high accuracy of short RNA-seq reads to correct the alignments of long RNA-seq reads by using –mix flag [40]. annotate_my_genomes using genome-guided StringTie-mix GTF assemblies reconstructed a 106-exon SSPO isoform, previously characterized with our merged short and long read alignments approach, including a c-terminal isoform of SSPO, previously presented in Fig. 2A (Figs 4A and 2A, respectively). The StringTie-mix approach also reconstructed a shorter SSPO isoform consisting in 89 exons, previously observed in illumina-alone assemblies (Supplementary Table 3, Fig. 2A). We noticed when we inputted the StringTie mix assembly into our pipeline, a shorter exon 1 is reconstructed in comparison with the merged short and long read alignment assembly. Still, both approaches assembled fifteen new aminoacids at the beginning of SSPO protein (Fig. 4B). Thus, exon 1 along with exon 2 confirms a spliced 5′UTR of the SSPO gene that encodes a larger isoform than the previously described for chicken SSPO, consisting of 106 exons and 5270 aminoacid, instead of 105 exons and 5255 aminoacid previously described for SSPO [43]. (Supplementary Table 3). The existence of these fifteen new aminoacids in chicken SSPO gene was further confirmed by protein blast analysis against vertebrate database and the COBALT tool (constraint-based alignment tool for multiple protein sequences) [47]. We detected four members of the *Aves* class presenting an SSPO isoform harboring this peptide at the beginning of the protein (Fig. 4C). These results suggest the 106-exon SSPO isoforms are correctly assembled and independently validated from other sources.

**Figure 5:**
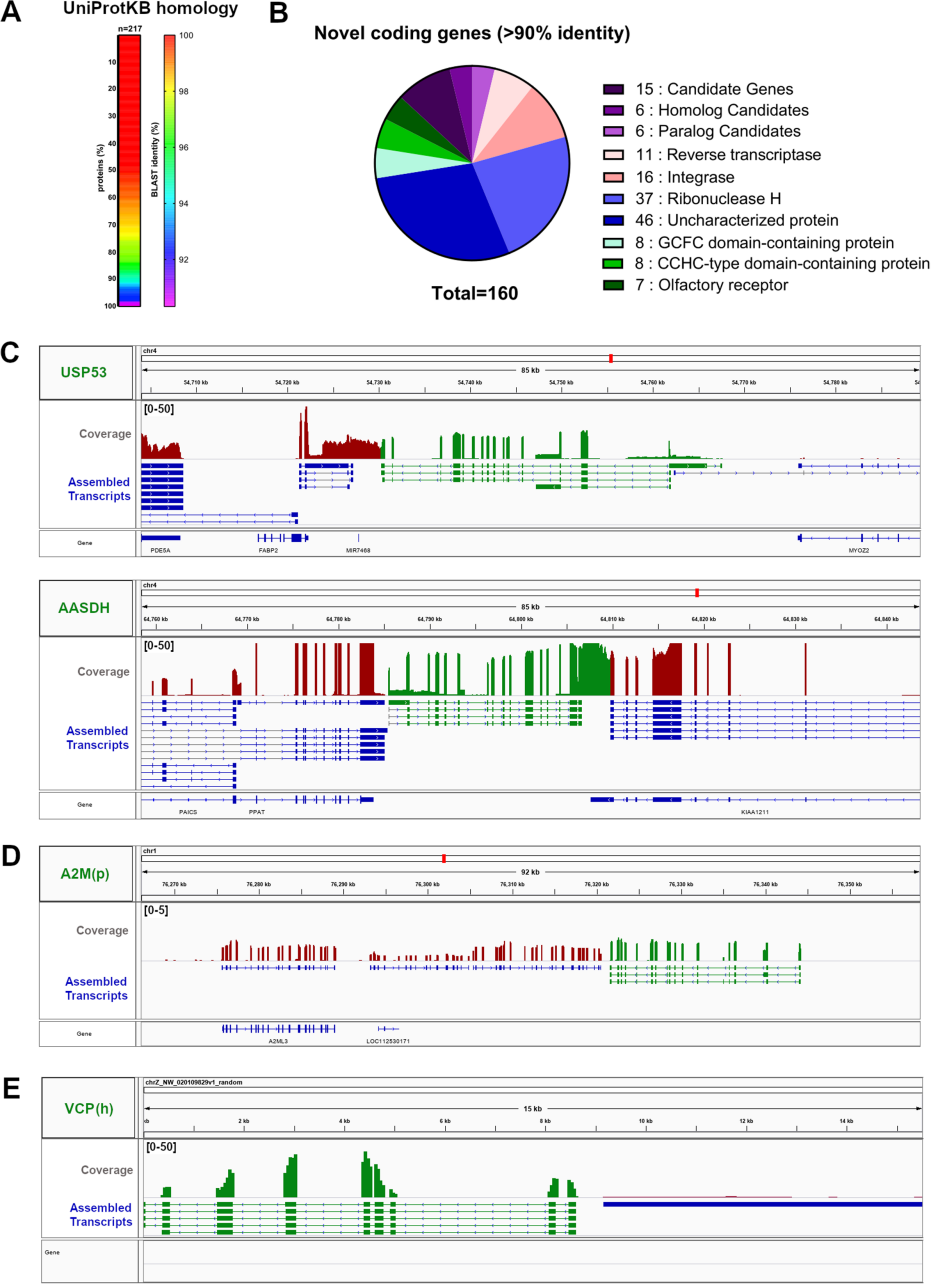
Homolog and paralog assignments successfully mapped missing genes in chicken reference annotations. **A**) BLASTp identities (in percentage) obtained by blasting the deduced proteome from the assembled transcriptome against all *Gallus gallus* proteome consisting in 34 730 uniprot chicken proteins (taxid 9031, https://www.uniprot.org/taxonomy/9031). Colors depict the BLAST identity percentage. **B**) Pie plot of 163 missing proteins displaying 90–100% identity with UniProt proteins. **C-E**) Missing genes in galGal6 reference, discovered by our pipeline. We denoted in green the candidate gene coverage including the assembled transcripts while we denoted in red the coverage of neighbor genes. (C) USP53 paralog, **D**) A2M paralog in chicken genome assigned to chromosome 1. **E**) Novel VCP homolog discovered in an unplaced contig belonging to chromosome Z.

To investigate if annotate_my_genomes pipeline can maintain the quality of the StringTie GTF inputs, we compared the F1 scores from the raw and the pipeline-processed GTF annotations from 1) de novo StringTie assembly from merged PacBio and Illumina alignments 2) genome-guided StringTie assembly from merged PacBio and Illumina alignments and 3) genome-guided StringTie mix assembly, respectively. In real datasets, annotate_my_genomes pipeline improved intron chain, transcript, and locus level of *de novo* StringTie raw annotations from *Gallus gallus, Mus musculus, Homo sapiens* and *Danio rerio* datasets, respectively. Also, the use of StringTie-mix assembly as input for our pipeline led to superior F1 scores with respect to de-novo and merged PacBio+Illumina StringTie assemblies (Fig. 4D). Overall, our pipeline did not sacrifice F1 score qualities from StringTie raw annotations and tend to maintain it or even improve it.

Finally, we benchmarked simulated datasets as well as real datasets, including C. elegans dataset (Fig. 4E). As in Fig. 4D and based on the F1 score, genome-guided StringTie-mix assemblies coupled with annotate_my_genomes slightly outperformed the genome-guided StringTie assembly derived from merged short and long read alignments and coupled with the pipeline. Sometimes the latter approach surpassed StringTie-mix at, intron chain, transcript and locus level, respectively. Again, annotate_my_genomes pipeline did not sacrifice F1 scores from raw StringTie annotations. Of note, StringTie mix coupled with our pipeline in genome-guided mode, obtained better F1 scores at all levels, across all species when compared with the other methods (Fig. 3D and E versus Fig. 4D and E, respectively).

In summary, with the availability of a good genome assembly including genome annotation in GTF format, it is beneficial to run annotate_my_genomes using a genome-guided StringTie GTF file as input when dealing with hybrid RNA-seq datasets, using either merged PacBio/Illumina merged alignments or the StringTie-mix approach. Also, in the absence of a genome annotation file in GTF format, it is worth running annotate_my_genomes, using de-novo StringTie GTF as input, since this method performs similar or sometimes better than BRAKER2/TSEBRA.

### Homolog assignments successfully mapped missing genes in chicken reference annotations

Next, we sought to use 34814 UniProt chicken protein sequences (taxid 9031, https://www.uniprot.org/taxonomy/9031, March 2022) to blast all deduced proteins from non-annotated coding genes in the assembled SCO transcriptome. Out of 499 novel proteins in the assembled SCO transcriptome, 217 proteins were present with >90% homology with UniProt chicken proteins and of them, around 70% were present with high similarity between the predicted and UniProt annotated proteins, demonstrating a good agreement between real and predicted protein sequences derived from our assembly process (> 98%, Fig. 4A). With this schema, we mapped missing paralogs in NCBI galGal6 genome by selecting proteins between 90–100% identity with *Gallus gallus* UniProt proteins. Since the genomic positions of the transcripts that encode for all these proteins are known, if two proteins have near 100% identity by blast analysis and the correspondent transcripts map within a 500 kb window, these proteins are considered as paralog candidates. Conversely, if the transcript maps to different loci positions, we considered them as homologs [48, 49]. Also, we examined if the transcripts that were associated with novel proteins overlapped with loci of previously annotated genes that were missing in the current annotation due to lack of evidence in the NCBI database. If that is the case, we considered these proteins as isoforms from missing genes.

Additionally, we benchmarked our blast results with the use of eggNOG-mapper, a method employing sequence homology search on a metagenomic scale [50, 51]. The output from eggNOG-mapper was intersected with the previous BLASTp results as described here: https://github.com/cfarkas/annotate_my_genomes/wiki#5-annotate-and-identify-homologs-in-novel-proteins-from-transcriptome. With both methods, we confirmed a substantial amount of novel coding genes encoding for endogenous retrovirus genes (ERVs) and genes containing homology with Ribonuclease H domains, zinc finger domains (CCHC domain-containing proteins), and olfactory genes, among others (Fig. 4B, Supplementary Table 3). Among the latter, we mapped fifteen missing candidate genes with different unfinished annotation status in NCBI database, including six novel homologous genes and novel paralog genes in chicken, respectively. Of the missing genes, Ubiquitin Specific Peptidase 53 (USP53) was absent in NCBI annotation but was present in the Ensembl annotation whereas Aminoadipate-Semialdehyde Dehydrogenase (AASDH) was missing in both databases (Fig. 4C, upper and lower, respectively). We also confirmed the existence of an alpha macroglobulin paralog, downstream to A2ML3 loci, located in chromosome 1 of galGal6 genome (Fig. 4D) and a novel homolog of VCP gene, spanning half of an unmapped contig belonging to chromosome Z (chrZ_NW_020109829v1_random, Fig. 4E). Since the VCP gene maps to chromosome Z, this novel homolog could be classified as a VCP paralog due to its close blast homology with VCP proteins, but the proximity of this unplaced contig cannot be determined with respect to the VCP gene. (Supplementary Table 3).

In summary, our ortholog/paralog assignations of non-annotated coding genes can help to increase annotation of important missing genes and aid to reconcile current annotations instead of choosing a single annotation tool from either NCBI, Ensembl, and/or other sources, a common practice in next-generation sequencing analysis [52]. Also, these procedures can aid to identify novel ERVs, possibly encoding for functional proteins due to their evolutionary conservation in vertebrates [53].

## Discussion

Here, we have developed and presented a hybrid RNA-seq annotation pipeline that helps to increase genomic annotation and allows researchers to discover missing/homologous genes by integrating previous genomic annotations in various animal genomes, providing a reconciled annotation in GTF format. This pipeline can be used for any organism that has an assembled genome and an NCBI available annotation in GTF format and relies on the use of StringTie as a transcript assembler. By using previously well-known tools, this pipeline can efficiently identify non annotated genes versus reconciled genes and distinguish between coding and non-coding genes. We benchmarked our pipeline against well-known bioinformatic genome annotation pipelines such as BRAKER1/2, TSEBRA and AUGUSTUS across real transcriptomes from five different species. According to the F1-score, using StringTie de-novo assemblies, our method performed equal or better than existing pipelines in terms of assembly quality. Moreover, F1-scores from our pipeline using as input StringTie assemblies from merged alignments and/or from the novel StringTie mix method, were the highest F1 values. To overcome coverage and noise variability across the employed dataset for benchmarking, we performed the same benchmarking using simulated transcriptomes across the five species. The latter lead to more dramatic results, where annotate_my_genomes coupled with StringTie assemblies with or without genome as guide, outperform all methods in all evaluated parameters. Therefore, if curated genome annotations are present for a given genome, it is beneficial to run our pipeline, since our method reconcile the current gene annotation and identify novel loci, without sacrificing F1-scores from the raw StringTie annotations. As proof of a concept, we fully assembled the chicken SSPO gene consisting of 106 exons rather than the previously published 105 exons, including the assembly of fifteen novel aminoacids at the N-terminal of SSPO protein. The latter, demonstrates good functionality in well-annotated genomes such as the chicken genome. Our pipeline assembled five transcripts with coding protein potential derived from the SSPO locus in the SCO. The assembly contained > 100 exon isoforms that are consistent with the presence of high molecular weight bands in the SCO previously reported by western blot using anti SCO-spondin (350-300 kDa) as well as lower bands ranging from 200 to 50 kD, probably corresponding to these smaller isoforms [28]. At the time of writing of this manuscript, the protein sequence of chicken SSPO was recently updated in NCBI (NCBI Reference Sequence: NM_001006351.3, March 09, 2022), the sequence associated with the novel chicken genome assembly bGalGal1.mat.broiler.GRCg7b (assembly accession: GCF_016699485.2). The SSPO protein from bGalGal1 assembly contains the mentioned fifteen residues described in this manuscript, which was only detected with annotate_my_genomes method and confirmed by protein homology in other four *Aves* class members. Therefore, these independent results support the quality of the SSPO transcripts assembled with our pipeline.

Regarding smaller assembled SSPO isoforms, both Illumina-alone and StringTie-mix approaches assembled an 89 and 25-exon isoforms (the latter, a c-terminal isoform). We did not discard the existence of these SSPO isoforms, but the protein products from these transcripts remains to be validated.

Since genome assemblies often update, this tool can aid in rapidly assigning genomic coordinates to missing genes, by inputting the updated genome assembly and correspondent annotation in the pipeline. This was the case of USP53 and AASDH genes, the latter was missing in all genomic annotations since the galGal4 chicken genome assembly was released in 2004 [54]. Also, we discovered a novel VCP homolog spanning half of an unmapped contig belonging to chromosome Z. We thus encourage researchers in the transcriptomics field to consider performing our novel assembly and re-annotation of RNA-seq data rather than using a single GTF annotation file in their studies. Importantly, in SCO organ development we discovered a myriad of divergent lncRNAs according to *FEELnc* lncRNA classification tool, potentially important in the differentiation of neural stem cells [55]. Overall, we propose that this pipeline will be a useful resource for obtaining a comprehensive view of the transcriptional landscape in each study and will help researchers to characterize novel transcriptomes and increase current genome annotations.

## Potential Implications

The present work will have two major impacts on the research community. On one side, our pipeline will facilitate the transcriptomic annotation of hybrid sequencing for research without advanced coding skills. This pipeline is implemented as an easy-to-use package on Anaconda/NextFlow/Docker platforms that integrates gold standard methods associated with transcriptome annotation. On the other side, our work advances our understanding of the chicken brain transcriptome by displaying an updated annotation, which includes full-length transcripts with challenging structures to assemble. We expect that our method will be useful for biologists interested in improving transcriptome annotation on a wide range of species, tissue and research areas. As well, our dataset will help to understand the development of specific brain structures providing a transcriptomic resource that can be consulted by all the community.

## Methods

### RNA isolation and qPCR

We dissected and pooled SCOs from outbred Gallus gallus embryos at Hamburger-Hamilton (HH) stages HH23 and/or HH30 in cold Phosphate Buffered Saline (PBS) solution. Total RNA was isolated using the RNeasy Mini Kit (QIAGEN). The concentration and quality of RNA were measured using Qubit™ RNA HS Assay Kit (Catalog number: Q32852). For qPCR reactions, we reverse transcribed up to 2 µg of RNA with M-MLV reverse transcriptase (PROMEGA) using 0.25 μg of Anchored Oligo(dT)20 Primer (Invitrogen, Catalog number: 12 577 011). All assayed primers in qPCR reactions are depicted in Supplementary Table 4. We performed qPCR reactions using KAPA SYBR FAST qPCR Master Mix (2X) Kit (Kapa Biosciences) with primer concentrations of 0.4 μM. For all PCR reactions, we used as cycling conditions an initial denaturation at 95°C for 3 min, then 40 cycles with 95°C for 5 s for denaturation and 60°C for 20 s of annealing/extension. The melting curve indicates no amplification of unspecific products.

### RNA sequencing

We assessed the integrity of five RNA samples from SCO HH23 (n = 2) and HH30 (n = 3), each one derived from at least 25 pooled animals coming from three different egg layings, by capillar electrophoresis (Agilent 2100 Bioanalyzer), obtaining RIN values between 8.8–9.5 per sample. Four PacBio RSII Isoform libraries were constructed by using 2 µg of total RNA from HH23 (n = 2) and HH30 (n = 2) SCO (Cold Spring Harbor Laboratory, Genomic Platform, USA). Sequencing was performed by using IsoSeq protocol (Pacific Biosciences) with long (>4 kb) and standard library enrichment sizes per stage. TruSeq Illumina libraries were prepared (two replicates by sample) and sequenced on a NextSeq Paired-End 150 bp middle output (Cold Spring Harbor Laboratory, Genomic Platform, USA). TruSeq Illumina libraries were prepared (two replicates by sample) and sequenced on a NextSeq Paired-End 150 bp middle output (Cold Spring Harbor Laboratory, Genomic Platform, USA).

### Isoform Assembly and gene annotation with annotate_my_genomes pipeline

We aligned PacBio reads against *Gallus gallus* genome (galGal6 version, GenBank assembly accession GCA_000002315.5) using *minimap2* aligner [30] obtaining depths of ∼ 34x. Illumina reads were automatically trimmed using fastp tool [31] and aligned against the referred Gallus gallus genome using HISAT2 aligner (RRID:SCR_015530)[32], obtaining depths of ∼ 10x. We sorted, assessed depth, and indexed bam files with *SAMtools* (RRID:SCR_002105)[56]. We merged the resulting BAM files from PacBio and Illumina read alignments into a single BAM file and we assembled transcripts from the latter alignment file using *StringTie* [57, 58] program with settings: -p 1 -j 2 -c 2 -v -a 4 for merged PacBio+Illumina assemblies or using –mix flag for StringTie mix assembly, respectively. We input assembled transcripts in GTF format to the *annotate_my_genomes* pipeline (https://github.com/cfarkas/annotate_my_genomes), obtaining coding/noncoding annotations and reconciled GTF file with current UCSC/NCBI genome annotation references. To reconcile transcripts and correspondent genes with reference genome annotations, the pipeline involves the use of standard UNIX tools, BEDtools [59], and GFF utilities [46] for GTF/GFF3 manipulations. First, we obtained transcripts from the input GTF file using *gffread* [46], and we used the GAWN pipeline (https://github.com/enormandeau/gawn) to initially annotate all possible proteins from the resulting transcripts using Blastx [60] and the Swissprot database, setting the following parameters: -evalue 1e-5 -qcov_hsp_perc 10 [61]. In these steps, the chicken genome (galGal6 genome) was indexed using the *GMAP aligner* [62]. Then, we assessed long noncoding RNAs training *FEELnc* classifier [34] with known coding RNAs from chicken (“NM_” prefix transcripts). Once lncRNAs were classified, the remaining unclassified transcripts were assessed to predicted coding regions and deduced proteins using *TransDecoder* gene prediction program (https://github.com/TransDecoder/TransDecoder). In this setting, we obtained coding genes, long-noncoding RNAs, and other genes (not classified as coding nor long-noncoding) merged in a single GTF file, indicating known genes by its USCS/NCBI symbol and novel genes with a “STRG” prefix. The installation and execution of annotate_my_genomes pipeline can be achieved through the Conda package manager (https://conda.io) [63], as a self-contained pipeline via the Nextflow workflow framework (https://www.nextflow.io/) [64], or via Docker (https://www.docker.com/), using a ready-to-use image that contains all requirements to implement the pipeline.

We visualized BAM files including annotated GTF files with IGV viewer [65]. We plotted GTF files from Fig. 3 using standalone pyGenomeTracks python package, available here: https://github.com/deeptools/pyGenomeTracks [66]. In order to calculate the Ex90N50 metric, we used a custom script that uses Salmon program [67]. Finally, we used *BUSCO tool* (RRID:SCR_015008)[68] to assess transcriptome completeness of PacBio and Illumina individual or combined assemblies.

## Pipeline Benchmarking

We downloaded, installed, and ran BRAKER1, BRAKER2, TSEBRA and AUGUSTUS pipelines along with our method using as inputs the following datasets: our *Gallus gallus* (galGal6) long and short read RNA-seq of the subcommissural organ (European Nucleotide Archive accession numbers PRJEB36569 and PRJEB36584, respectively), *Homo sapiens* (hg38) long-read cDNA sequencing of HAP1 cells (NCBI BioProject PRJNA673144), *Mus musculus* (mm10) long read sequencing of preimplantation embryo transcriptome (NCBI BioProject PRJNA577068), *Danio rerio* (danRer11) long-read sequencing transcriptome during zygotic genome activation (NCBI BioProject PRJNA395690) and *Caenorhabditis elegans* (ce11) nanopore-only direct-RNA sequencing across larvae development (European Nucleotide Archive accession number PRJEB31791) datasets, respectively. We also simulated *Caenorhabditis elegans* Illumina datasets SRR14682986 from L1 larvae cells to fulfill hybrid dataset comparison across species (BioProject: PRJNA733501). The dataset acquisition, preprocessing, genomic alignments and pipeline executions are described in detail here: https://github.com/cfarkas/annotate_my_genomes/wiki/annotate_my_genomes-benchmarking. The precision, recall, and their harmonic mean—the F1-score—as measures of gene prediction accuracy were obtained by using gffcompare and are available in Supplementary Table 2.

### Dataset simulation

We simulated PacBio reads using IsoSeqSim tool (https://github.com/yunhaowang/IsoSeqSim). For these purposes, we used as inputs the genome (FASTA format) and the NCBI reference GTF from each of the referred species, respectively. We simulated substitutions (mismatches), deletions and insertions up to 1.731%, 1.090% and 2.204%, respectively, as observed in the Alzheimer's disease brain Iso-Seq data released by PacBio in 2016: (https://downloads.pacbcloud.com/public/dataset/Alzheimer2019_IsoSeq/) and we established the average read count per transcript to 20 (–nbn parameter). In the same manner, we also simulated illumina reads using reference transcriptome for each species (FASTA format) using ReSeq tool [69].

For the latter, we mapped the real illumina reads datasets employed in the benchmarking from each species against their reference transcriptome (in FASTA format) using *bowtie2* aligner with the parameter -X equals to 2000 [70], we sorted the resulting BAM files and we inputted the resulting aligned BAM files along with the reference transcriptome (in FASTA format) from each species to simulate paired illumina datasets per species. In each case, we simulated illumina reads using ReSeq illuminaPE mode using the parameters -j 50 and -c 20, respectively.

After these steps, PacBio and Illumina simulated datasets were aligned against their reference transcriptomes using *minimap2* (using -ax splice flag) and *HISAT2* aligners (RRID:SCR_015530) on default mode, respectively and the resulting simulated transcriptomes were merged and assembled using *StringTie* or inputed for *StringTie-mix* assembler (RRID:SCR_016323). The resulting GTF files were inputted into annotate_my_genomes pipeline to obtain annotated GTF files while the individual Illumina and PacBio alignments in BAM format were used as inputs for BRAKER1/2 and TSEBRA pipelines, respectively. The precision, recall, and their harmonic mean (F1-score) were obtained by using gffcompare and are available in Supplementary Table 2.

## Homolog Assignments

To assess possible homologs in novel coding genes (cds), we blasted the novel predicted proteins from these cds against the UniProt *Gallus gallus* proteome (taxid 9031) [71] with the setting -max_hsps 1 -max_target_seqs 1 in blastp command [72]. Then, we parsed these results and compared the genomic positions of all novel protein matches against the genomic positions of proteome indexed in NCBI. If two matches with 90–100% homology were found within the same loci (<0.5 Mb), we considered them as paralogs [48, 49]. Otherwise, we considered these matches as missing genes in the reference annotation. We also integrated to the previous results the metagenome-level annotation of novel proteins using eggNOG-mapper ortholog classification software [50, 51]. All relevant commands to reproduce these analyses are available here: https://github.com/cfarkas/annotate_my_genomes/wiki#5-annotate-and-identify-homologs-in-novel-proteins-from-transcriptome

### Immunohistochemistry

Immunohistochemistry was performed following the protocol described in Vera et al. (2013), using anti-NCAM cytoplasmic domain antibody (4D from Developmental Studies Hybridoma Bank, University of Iowa, Iowa City, IA) as well as with a rabbit anti Reissner's fiber glycoproteins antibody (AFRU) that recognizes SCO-spondin [28]. As second antibodies we used Goat anti-mouse Alexa-546 and anti-rabbit Alexa-488 antibodies (Invitrogen, Carlsbad, CA), and nuclei were visualized with TOPRO-3 (Invitrogen, Carlsbad, CA).

## Additional Supplementary Files

**Supplementary Figure 1:** Illumina sequencing coverage at *SSPO* locus (chr2:466581–503024 in galGal6 assembly) of whole-brain RNA extracted at stages HH31 and HH36, respectively (see tracks with green names). Illumina sequencing coverage at *SSPO* locus (chr2:466581–503024 in galGal6 assembly) of SCO RNA extracted at stages HH23 and HH30, respectively (see tracks with blue names). Tracks were obtained from galGal6 UCSC genome browser (https://genome.ucsc.edu/).

**Supplementary Figure 2**: **A**) Coverage plots of all assembled SSPO isoforms found with the pipeline, obtained with RSEQC. Each line color denotes the coverage of each Illumina sequencing across the gene body percentiles (V41.sorted = HH23_rep1, V42.sorted = HH23_rep2, V71.sorted = HH30_rep1 and V72.sorted = HH30_rep2). **B)** Same as A) just considering 5′ and 3′ end SSPO isoforms (transcripts N°STRG.7690.2 and STRG.7690.5, see Fig. 2).

**Supplementary Figure 3**: CPU time executions of the different assayed methods across RNA-seq datasets employed in this study. AmyG = annotate_my_genomes.

**Supplementary Table 1**: Annotation of the assembled transcriptome with hybrid sequencing technologies, including predicted long-noncoding RNAs. Reference Transcript annotation sheet contains StringTie transcripts intersections with NCBI reference transcripts, including correspondent transcript sequences (n = 61 679). Novel Transcript Annotation sheet contains novel transcripts including correspondent transcript sequences (n = 5610). Candidate lncRNA classes contain all transcripts classified as lncRNAs by *FEELnc* software.

**Supplementary Table 2**: F1-scores, calculated with precision and recall values from gffcompare across real and simulated RNA-seq datasets employed in this study. Precision and recall values were obtained by comparing base, exon, intron, intron-chain, transcript, and locus level between output GTF from several genome annotation pipelines, and the reference GTF annotation from NCBI (considered as truth).

**Supplementary Table 3**: SSPO transcript and protein annotations obtained with StringTie (PacBio+Illumina merged approach) and StringTie-mix

**Supplementary Table 4**: Ortholog and paralog identification within novel *Gallus gallus* proteins. **Sheet 1** contains eggNOG-mapper ortholog annotations from novel proteins. **Sheet 2** contains blastp results from novel proteins against Gallus Gallus uniprot database (taxid 9031). The latter results were filtered with a cut-off between 90–100% identity. **Sheet 3** contains the intersection between the refereed datasets. **Sheet 4** contains manual annotation of the latter intersection, respectively.

**Supplementary Table 5**: List of publicly available sequencing datasets and primers used in this study.

giac099_GIGA-D-22-00061_Original_Submission

giac099_GIGA-D-22-00061_Revision_1

giac099_Response_to_Reviewer_Comments_Original_Submission

giac099_Reviewer_1_Report_Original_Submission Roberto Vera Alvarez, Ph.D -- 4/8/2022 Reviewed

giac099_Reviewer_2_Report_Original_Submission Andrey D. Prjibelski, M.Sc. -- 4/21/2022 Reviewed

giac099_Reviewer_2_Report_Revision_1 Andrey D. Prjibelski, M.Sc. -- 8/23/2022 Reviewed

giac099_Supplemental_Files

## Availability of source code and requirements

Project name: annotate_my_genomes (version 3.3, March 2022)

Project home page: https://github.com/cfarkas/annotate_my_genomes [73]

Operating system(s): Ubuntu/MacOSX

Programming language: BASH, Python, NextFlow, Docker

Other requirements: ncbi-blast+ version equal or higher than v2.7.1, SAMtools and Python3.

License: MIT License

RRID:SCR_022922

## Data Availability

All computational steps to replicate the analysis performed in this paper are available here: https://github.com/cfarkas/annotate_my_genomes [73]. We provide on the GitHub page an easy-to-install package of our pipeline that can be run on a modern laptop using Linux/Ubuntu operating system. PacBio and Illumina RNA-sequencing datasets are available at European Nucleotide Archive (ENA) Accession Number PRJEB36569 (PacBio) and PRJEB36584 (Illumina). Snapshots of our code and other data further supporting this work are openly available in the *GigaScience* repository, GigaDB [74].

## Abbreviations

AASDH: Aminoadipate-Semialdehyde Dehydrogenase
CCS: circular consensus sequences
ERVs: Endogenous retrovirus genes
GTF: Gene Transfer Format
LncRNA: long non-coding RNAs
NGS: Next Generation Sequencing
ON: Oxford Nanopore
RNA-seq: RNA-sequencing
SCO: sub-commissural organ
SMRT: single-molecule real-time
SSPO: SCO-spondin:
USP53: Ubiquitin Specific Peptidase 53
VCP: Transitional endoplasmic reticulum ATPase
HH: Hamburger-Hamilton

## Competing interests

The authors declare that they have no competing interests

## Funding

This work was supported by Fondo Nacional de Desarrollo Científico y Tecnológico, FONDECYT [1191860 to T.C] and FONDECYT de Iniciación [11190401 to ETS]. CF and JJH received partial funding from the CIHR and CancerCare Manitoba Foundation.

## Authors’ Contributions

TC and CF: conceived and designed the experiments. CF, AR: were involved in sample preparation, qPCR, and IHC. CF, ETS, and CDH: were involved in data analysis and figure processing. CF, AM and MGO implemented the pipeline in both Anaconda and NextFlow environments. CF, TC, JJH, ETS: were involved in results discussion and paper writing.

## Ethical Statement

The ethics, bioethics and biosafety committee of Vicerrectoría de Investigación y Desarrollo de la Universidad de Concepción has been reviewed the proposal of the project “PROYECTO Nº 1191860,” adjudicated from CONCURSO FONDECYT REGULAR 2019, entitled “SCO-SPONDIN: A CEREBROSPINAL FLUID MATRICELLULAR PROTEIN FULFILLING CRUCIAL NEUROGENIC FUNCTIONS” proposed by the principal investigator DRA. TERESA CAPRILE ELOLA-OLASO, ascribed professor at Departamento de Biología Celular de la Facultad de Ciencias Biológicas de la Universidad de Concepción, has confirmed that fulfill the national and international established norms, ethical and bioethical principles and biosafety procedures regarding animal handling and experimentation (in this case, Gallus gallus embyos), including the appropriate handling of chemical biological waste derived from this proposal. The approval of this document is follows gudelines of National law Res. Exenta Nº 157, del 24 de enero de 2013 from CONICYT. Reference document: CEBB 408–2019.
