# Supplementary material for: annotate_my_genomes: an easy-to-use pipeline to improve genome annotation and uncover neglected genes by hybrid RNA sequencing
Source: Gigascience. 2022 Dec 6;11:giac099. doi: 10.1093/gigascience/giac099 (PMC9724561; doi:10.1093/gigascience/giac099)
Supplement: giac099_GIGA-D-22-00061_Original_Submission [file giac099_giga-d-22-00061_original_submission.pdf]

GigaScience

annotate_my_genomes: an easy-to-use pipeline to improve genome annotation and uncover neglected genes by hybrid RNA sequencing.

--Manuscript Draft--

Manuscript Number:	GIGA-D-22-00061
Full Title:	annotate_my_genomes: an easy-to-use pipeline to improve genome annotation and uncover neglected genes by hybrid RNA sequencing.
Article Type:	Research
Funding Information:	FONDECYT (1191860) — Dr Teresa Caprile<br>FONDECYT INICIACIÓN (11190401) — Dr Estefanía Tarifeño-Saldivia
Abstract:	Abstract<br><br>Background: The advancement of hybrid sequencing technologies is increasingly expanding genome assemblies that are often annotated using hybrid sequencing transcriptomics, leading to improved genome characterization and the identification of novel genes and isoforms in a wide variety of organisms.<br>Results: We developed an easy-to-use genome-guided transcriptome annotation pipeline that uses assembled transcripts from hybrid sequencing data as input and distinguishes between coding and long non-coding RNAs by integration of several bioinformatic approaches, including gene reconciliation with previous annotations in GTF format. We demonstrated the efficiency of this approach by correctly assembling and annotating all exons from the chicken SCO-spondin gene (containing more than 105 exons), including the identification of missing genes in the chicken reference annotations by homology assignments.<br>Conclusions: Our method helps to improve the current transcriptome annotation of the chicken brain. Our pipeline, implemented on Anaconda/Nextflow is an easy-to-use package that can be applied to a broad range of species, tissues, and research areas helping to improve and reconcile current annotations. The code and datasets are publicly available at https://github.com/cfarkas/annotate_my_genomes
Corresponding Author:	Teresa Caprile, Ph.D.<br>Universidad de Concepción: Universidad de Concepcion<br>Concepción, Bío-Bío CHILE
Corresponding Author Secondary Information:	
Corresponding Author's Institution:	Universidad de Concepción: Universidad de Concepcion
Corresponding Author's Secondary Institution:	
First Author:	Carlos Farkas, Doctor
First Author Secondary Information:	
Order of Authors:	Carlos Farkas, Doctor
	Antonia Jesus Recabal, Doctor
	Andy Mella, Master's
	Daniel Candia-Herrera, Master's
	Maryori González Olivero, Bachelor's
	Jody Jonathan Haigh, Doctor
	Estefanía Tarifeño-Saldivia, Doctor
	Teresa Caprile, Doctor
Order of Authors Secondary Information:	

Powered by Editorial Manager® and ProduXion Manager® from Aries Systems Corporation

Additional Information:	
**Question**	**Response**
Are you submitting this manuscript to a special series or article collection?	No
**Experimental design and statistics**<br><br>Full details of the experimental design and statistical methods used should be given in the Methods section, as detailed in our Minimum Standards Reporting Checklist. Information essential to interpreting the data presented should be made available in the figure legends.<br><br>Have you included all the information requested in your manuscript?	Yes
**Resources**<br><br>A description of all resources used, including antibodies, cell lines, animals and software tools, with enough information to allow them to be uniquely identified, should be included in the Methods section. Authors are strongly encouraged to cite Research Resource Identifiers (RRIDs) for antibodies, model organisms and tools, where possible.<br><br>Have you included the information requested as detailed in our Minimum Standards Reporting Checklist?	Yes
**Availability of data and materials**<br><br>All datasets and code on which the conclusions of the paper rely must be either included in your submission or deposited in publicly available repositories (where available and ethically appropriate), referencing such data using a unique identifier in the references and in the “Availability of Data and Materials” section of your manuscript.	Yes

Powered by Editorial Manager® and ProduXion Manager® from Aries Systems Corporation

Have you have met the above requirement as detailed in our Minimum Standards Reporting Checklist?	

Manuscript

Click here to access/download;Manuscript;Manuscript-annotate_my_genomes_GigaScience_22032022.docx

Title: annotate_my_genomes: an easy-to-use pipeline to improve genome annotation and uncover neglected genes by hybrid RNA sequencing.

Carlos Farkas*[1], Antonia Recabal[2], Andy Mella[6,7], Daniel Candia-Herrera[3], Maryori González Olivero[2], Jody Jonathan Haigh[4,5], Estefanía Tarifeño-Saldivia*[3] and Teresa Caprile[2]*

1: Laboratorio de Investigación en Ciencias Biomédicas, Departamento de Ciencias Básicas y Morfología, Facultad de Medicina, Universidad Católica de la Santísima Concepción, Concepción, Chile.

2: Departamento de Biología Celular, Facultad de Ciencias Biológicas, Universidad de Concepción, Chile

3: Departamento de Bioquímica y Biología Molecular, Facultad de Ciencias Biológicas, Universidad de Concepción, Chile

4: CancerCare Manitoba Research Institute, Winnipeg, MB, Canada.

5: Department of Pharmacology and Therapeutics, Rady Faculty of Health Sciences, University of Manitoba, Winnipeg, MB, Canada

6: Instituto de Ciencias Naturales, Universidad de las Américas, Chile

7: Centro Integrativo de Biología y Química Aplicada (CIBQA), Universidad Bernardo O'Higgins, Santiago 8370854, Chile

***Corresponding authors of this study:**

Carlos Farkas (cfarkas@udec.cl), Estefania Tarifeño-Saldivia (etarisal@udec.cl) and Teresa Caprile (tcaprile@udec.cl)

Abstract

Background: The advancement of hybrid sequencing technologies is increasingly expanding genome assemblies that are often annotated using hybrid sequencing transcriptomics, leading to improved genome characterization and the identification of novel genes and isoforms in a wide variety of organisms.

Results: We developed an easy-to-use genome-guided transcriptome annotation pipeline that uses assembled transcripts from hybrid sequencing data as input and distinguishes between coding and long non-coding RNAs by integration of several bioinformatic approaches, including gene reconciliation with previous annotations in GTF format. We demonstrated the efficiency of this approach by correctly assembling and annotating all exons from the chicken SCO-spondin gene (containing more than 105 exons), including the identification of missing genes in the chicken reference annotations by homology assignments.

Conclusions: Our method helps to improve the current transcriptome annotation of the chicken brain. Our pipeline, implemented on Anaconda/Nextflow is an easy-to-use package that can be applied to a broad range of species, tissues, and research areas helping to improve and reconcile current annotations. The code and datasets are publicly available at https://github.com/cfarkas/annotate_my_genomes

Contact: cfarkas@udec.cl; etarisal@udec.cl; tcaprile@udec.cl

Keywords: Transcriptome annotation, Genome Annotation pipeline, SCO-spondin, hybrid sequencing

Background

The emergent advancement of Next Generation Sequencing (NGS) combined with novel genome assembly methods greatly improved genome characterization, identifying novel genes and isoforms in both model as well as non-model organisms [1-3]. RNA-sequencing (RNA-seq) based on short reads resolve transcriptomes in a limited manner due to technical limitations in assembly [4]. Long-read RNA-seq technologies alone or combined with short-read sequencing often improve the quality and contiguity of transcriptome assemblies [5, 6]. Long-read technologies such as PacBio single-molecule real-time (SMRT) and Oxford Nanopore (ONT) sequencing technologies (hereafter PacBio and Nanopore sequencing, respectively) are more efficient than short-read RNA-seq to reconstruct full-length transcripts by using error correction and polishing pipelines [7]. Well-established PacBio-only based pipelines such as IsoSeq [8, 9] and IsoCon [10] often perform well on these tasks and hybrid sequencing even outperforms these methods producing better transcriptome assemblies [11-13]. After assessing the best transcriptome assembly with tools such as rnaQUAST [14], SQANTI [15], or by using multiple assemblies to improve gene structure annotation [16], an additional challenge in transcriptomic studies is the feature identification and annotation process. Initially, pipelines such as MAKER integrated trained *ab initio* gene predictions, Expressed Sequence Tags (EST), and proteins to annotate genes from a given genome [17, 18]. In the same way, the gene prediction program AUGUSTUS accurately predicts genes using supervised training of EST and proteins as external hints, including the use of short read RNA-seq alignments to improve final gene prediction [19, 20]. Later, BRAKER1 pipeline was developed, a short read RNA-seq genome annotation pipeline that combines AUGUSTUS and GeneMark-ET, an unsupervised RNA-seq gene prediction tool [21, 22]. Subsequently, BRAKER2 improved BRAKER1 work by integrating iterative-training gene predictions from

GeneMark-ET and AUGUSTUS, transcriptomic data, and external protein support altogether [23]. More recently, the TSEBRA pipeline selects transcripts from BRAKER1 and BRAKER2 predictions altogether, by ranking all transcript predictions according to the RNA-seq and homologous protein evidence support and selecting the best candidates [24]. Evidence-based proteomics and transcriptomics for gene-finding, offers complete and reliable genome annotations, but dedicated tools for hybrid RNA-seq analysis are also needed. Regarding the latter, the long-read annotation tool LoReAN combines Trinity-based transcript assemblies and BRAKER1 predictions from short-read RNA-seq, including clustered transcript reconstruction from long-read sequencing technologies and proteome data as well [25]. Although more effective than short-read annotation pipelines, the latter pipeline can be time and CPU consuming, especially in the use of Trinity assembly process when large datasets are employed. In the present work, we present annotate_my_genomes, an easy-to-use transcriptome annotation pipeline that uses assembled transcripts from hybrid sequencing data as input and distinguishes between coding and long non-coding RNAs (lncRNAs) by integration of several well-established approaches, including gene reconciliation with previous annotations. This method requires a reference genome as a guide and leads to superior transcriptome assembly and annotation when compared to traditional Illumina or PacBio RNA-seq protocols such as IsoSeq as well as similar pipelines [26, 27]. We demonstrated the efficiency of this approach by correctly assembling all exons from the chicken SSPO gene (containing more than 105 exons) and mapping missing genes in the chicken reference genome by homology assignments. We demonstrated that using StringTie GTF assembly as input, our method tends to improve the current genome annotation, surpassing BRAKER1/2 and TSEBRA performances. Importantly, the presented data provides the first transcriptional landscapes of sub-commissural organ (SCO) of the chick embryo, a brain gland related to different morphogenic events, such as the regulation of brain development and body axis alignment [28, 29].

Data Description

The transcriptome of the chick embryo sub-commissural organ (SCO) was performed using a combination of Illumina (short-reads) and PacBio (long-reads) sequencing. To prepare the samples, we dissected and pooled 25 SCOs from outbred Gallus gallus embryos at Hamburger-Hamilton (HH) stages HH23 and HH30. Total RNA was isolated using the RNeasy Mini Kit (QIAGEN). The concentration and quality of RNA were measured using Qubit™ RNA HS Assay Kit (RIN values between 8.8-9.5 per sample). Four PacBio RSII Isoform libraries were constructed by using 2 μg of total RNA from HH23 (n=2) and HH30 (n=2) SCO (Cold Spring Harbor Laboratory, Genomic Platform, USA). Sequencing was performed by using IsoSeq protocol (Pacific Biosciences) with long (>4kb) and standard library enrichment sizes per stage. TruSeq Illumina libraries were prepared (two replicates by sample) and sequenced on a NextSeq Paired-End 150 bp middle output (Cold Spring Harbor Laboratory, Genomic Platform, USA). PacBio and Illumina RNA-sequencing datasets are available at European Nucleotide Archive (ENA) Accession Number PRJEB36569 (PacBio) and PRJEB36584 (Illumina).

Analyses

Combined PacBio and Illumina RNA sequencing assembly improved gene annotation in the chicken transcriptome.

To uncover the transcriptome of chicken SCO organs at Hamburger-Hamilton (HH) stages HH23 and/or HH30stages, we performed a hybrid sequencing approach using long (>4kb) and standard library enrichment sizes for PacBio, and Illumina platforms. Instead of assembling PacBio reads by traditional pipelines such as IsoSeq [26, 27] and/or IsoCon [10], we aligned reads against the Genome Reference Consortium Chicken Build 6a (GRCg6a) reference genome (assembly GCF_000002315.5) by using *minimap2*, a splice aware aligner [30]. Illumina short reads were trimmed using fastp tool [31] and aligned using HISAT2 [32]. Posteriorly, the alignments from both technologies were merged. Transcripts were assembled from merged aligned reads using *StringTie* program and transcripts were annotated based on the NCBI/UCSC annotation associated with GRCg6a assembly (March 2018 version). Assembled transcripts in GTF format were used as input for our annotate_my_genomes pipeline. First, assembled transcripts were identified based on homology with BLASTX [33]. New transcripts (not included on the genome annotation) were further classified as coding or non-coding, using the long non-coding RNA classification tool *FEELnc* [34]. Non-lncRNA transcripts presenting a BLASTX match were further collected, and open reading frames were predicted by using *TransDecoder* gene coding prediction pipeline (https://github.com/TransDecoder/TransDecoder). Finally, we employed the UniProt database [35] to identify novel coding transcripts (**Figure 1A**). With this setting, we benchmarked the quality of these assemblies produced by every technology including the merged approach. Ex90N50 values from IsoSeq transcriptome assembly surpassed both merged and Illumina

transcriptome assemblies (**Figure 1B,** left). The merged assembly improved Illumina bases per sequencing and mappable assembled transcripts, suggesting an overall improvement of Illumina sequencing technology by merging assemblies from both technologies, while IsoSeq assembly surpassed both merged and Illumina assemblies (**Figure 1B,** middle and right, respectively).

Even though IsoSeq displayed better assembly statistics than Illumina technology, Illumina and merged sequencing technologies led to higher-quality assemblies assessed by the number of completed BUSCOs found in the *aves* lineage (aves_odb10) (**Figure 1C**). This result is expected since Illumina has better sequencing depth and quality than IsoSeq (~1% versus ~11% sequencing error, respectively) [36]. Also, the merged strategy slightly improved the completeness of the SCO transcriptome compared to the Illumina assembly (1%). Thus, despite higher Ex90N50 values of IsoSeq assembly, we further selected the merged strategy to annotate genes, because of its increased quality over Illumina and PacBio assemblies alone.

Our pipeline identified 19,690 reconciled genes including 4,292 candidate genes that are not annotated on the chicken GRCg6a reference genome, from them 64% and 9% corresponding to coding genes (**Figure 1D**, left and right, respectively). At the level of transcripts, we observed 61,940 reconciled and 5,201 non-annotated transcripts, where 76% and ~10% are coding transcripts, respectively (see **Figure 1E**, left and right, respectively, and **Supplementary Table 1**). Of note, a substantial number of transcripts not classified as either coding or long non-coding RNAs composed missing transcripts (65%), arguing that these transcripts survived the RNA quality control system removal from the cell [37, 38] and could correspond to small RNAs, incomplete gene models, and/or transcripts emanating from repeat regions [39]. We aimed to classify the discovered long noncoding RNAs (lncRNAs) by location and subtype, by using the FEELnc classification tool [34]. By location, concerning neighboring genes, we found a significant proportion of exonic/intronic lncRNAs types (~11%,

Figure 1F). Also, by orientation, a significant proportion of all lncRNAs are divergent lncRNAs (27%, **Figure 1G**). Overall, our results confirm that hybrid sequencing is beneficial for a comprehensive and reconciliated characterization of a given transcriptome, which agrees with a previous report [25]. In addition, our tool provides a way to streamline the annotation process in a user-friendly manner.

The Giant SSPO gene is fully reconstructed by hybrid sequencing technology.

We aimed to assemble and annotate with our pipeline the giant gene SSPO, a 105-exon gene encoding the main secreted glycoprotein that forms the Reissner fiber from the subcommissural organ [40, 41]. The SSPO gene in chicken is provisionally classified as a protein coding gene by NCBI (https://www.ncbi.nlm.nih.gov/gene/420367), thus, we challenged our hybrid assembly and annotation pipeline with this giant gene. Previous whole-brain Illumina sequencing at HH31/HH36 stages did not contain any mapped read to SSPO locus, probably because SCO is the unique source of SSPO expression (**Supplementary Figure 1**). The latter also explains the consistent absence of SSPO in coding reference annotations due to lack of SCO transcriptome data (see http://www.ensembl.org/Gallus_gallus/Gene/Summary?g=ENSGALG00000033417;r=2:467007-502923;t=ENSGALT00000058788). We examined the coverage of PacBio, Illumina, and the merge of HH23 and HH30 RNA sequencing, demonstrating that PacBio sequencing fails to properly assemble the SSPO gene (SCO-spondin), one of the main secreted glycoproteins from the SCO (**Figure 2A**). Also, SSPO related transcripts did not figure in the circular consensus sequences (CCS) or in the high- or low-quality assembled transcripts from IsoSeq (https://github.com/ben-lerch/IsoSeq-3.0). Conversely, Illumina sequencing from HH23/HH30 stages aligned to SSPO locus (see turquoise and blue colored tracks in **Figure**

2A, respectively). Combined Illumina-PacBio sequencing led to the assembly of four SSPO isoforms of 106, 95, 89, and 25 exons, respectively (see the red-colored track and gene track underneath in **Figure 2A**). The merged strategy led to the assembly of two SSPO isoforms of 106 exons, and 3 isoforms of 105, 17, and 25 exons, all of them classified to encode proteins and not lncRNAs (see transcripts 1, 3, 2, 4, and 5 in Gene_PacBio_Illumina track from **Figure 2A**, respectively). Transcript 1 encodes a protein of 5270 amino acids with 98.88% of identity with a previously deduced SSPO protein in chicken, derived from a cloned cDNA in SCO (GenBank accession AJ 866919) [42]. The latter protein contains 5255 amino acids encoded within 105 exons, lacking the first assembled exon of our reconstructed transcripts. Thus, the merged strategy leads to a superior assembly consisting of five alternative isoforms (see green-colored numbers indicating the new exons in **Figure 2A**). Also, the use of only Illumina assembly leads to an incomplete assembly of SSPO gene at the 5' end (see Illumina gene track in **Figure 2A,** the track called "merged_illumina.gtf"). Regarding the latter, no degradation of 5' or 3' ends of SSPO gene transcripts was detected after coverage inspection of SCO Illumina datasets with the RSeQC package [43] (**Supplementary Figure 2**). By using sets of primers designed at the beginning and the end of SSPO gene, we confirmed increased transcription at the 3' end of SSPO at stage HH30 in the SCO, supporting the existence of a C-terminal isoform (**Figure 2B**). Immunohistochemistry of SCO at stages HH23 and HH30 confirm increased expression of SCO at HH30 (**Figure 2C**), as previously confirmed by western blot [28]. In summary, hybrid sequencing leads to better assemblies by improving contiguity and lowering misassembles with the combination of PacBio long reads and high-quality Illumina short reads, respectively.

Pipeline Benchmarking

We initially inspected the assembly of SSPO gene in *Gallus gallus* genome (galGal6) using annotate_my_genomes and dedicated genome annotation pipelines such as SQANTI3 [15], BRAKER1 [21], BRAKER2 [23], including the recent TSEBRA method [24]. AUGUSTUS *ab initio* predictions were also included in the comparison [20]. BRAKER2, TSEBRA and annotate_my_genomes, but not *ab initio* AUGUSTUS and SQANTI3 methods correctly assembled all previously described exons from SSPO (n = 105) (**Figure 3A**). Of notice, annotate_my_genomes method assembled these 105 exons, including an additional exon (hereafter exon 1) across SSPO isoforms (**Figure 3B**). These preliminary results indicate that our method can resolve more exons than the referred methods, however this result might not necessarily imply a better assembly.

Therefore, we additionally annotated *Mus musculus* (mm10), *Homo sapiens* (hg38), *Danio rerio* (danRer11) and *Caenorhabditis elegans* (ce11) RNA-seq datasets sequenced with short and long reads, using the referred annotation methods (see **Supplementary Table 4** for sequencing datasets). We compared the gene annotation predictions from each method in GTF format against the NCBI reference GTF from each genome (the latter considered as truth), using gffcompare [44]. The evaluated parameters covered bases, exon, intron, intron-chain, transcript, and locus level assessments, as described here: https://ccb.jhu.edu/software/stringtie/gffcompare.shtml). For each evaluated parameter, gffcompare retrieved precision ($\frac{TN}{FP+TN}$, where TN = True Negatives and FP = False Positives, respectively) and recall ($\frac{TP}{FN+TP}$, where TP = True Positives and FN = False Negatives). Then, we calculated the harmonic mean of the latter values (F1-score) as follows: $\frac{2x(precision\ x\ recall)}{precision+recall}$. We considered F1-score as the final measure of gene prediction accuracy for each method. In

all evaluated parameters, annotate_my_genomes using genome-guided StringTie GTF as input outperform all methods in our sequencing dataset (galGal6) (**Figure 3C**, see asterisks). This behaviour was also seen in *Mus musculus*, *Homo sapiens*, *Danio rerio* and *Caenorhabditis elegans*, excepting at the intron level, where BRAKER2 outperform our method in three out of four datasets (**Figure 3D**, see asterisks). Of note, SQANTI3 outperformed our method in one evaluated parameter (locus level) in one dataset (*Danio rerio*). annotate_my_genomes using de novo GTF assembly produced with StringTie as input performed similarly as BRAKER2 or TSEBRA in each dataset, sometimes surpassing BRAKER2/TSEBRA (see galGal6 in **Figure 3C** and mm10, hg38 in **Figure 3D**, respectively).

We also noticed CPU times were equal or inferior when annotate_my_genomes method is employed, in comparison with BRAKER1, BRAKER2, or TSEBRA across the referred RNA-seq datasets (**Supplementary Figure 3**) Although SQANTI3 method was very fast, this method only took as input the alignment of long reads in the form of a GTF assembly, not considering short read processing, thus explaining the short runs.

In summary, with the availability of a good genome assembly including genome annotation in GTF format, it is beneficial to run annotate_my_genomes using a genome-guided StringTie GTF file as input when dealing with hybrid RNA-seq datasets. Also, in the absence of a genome annotation file in GTF format, it is worth running annotate_my_genomes, using de-novo StringTie GTF as input, since this method performs similar or sometimes better than BRAKER2/TSEBRA.

Homolog assignments successfully mapped missing genes in chicken reference annotations.

Next, we sought to use 34,814 UniProt chicken protein sequences (taxid 9031, https://www.uniprot.org/taxonomy/9031, March 2022) to blast all deduced proteins from non-annotated coding genes in the assembled SCO transcriptome. Out of 499 novel proteins in the assembled SCO transcriptome**,** 217 proteins were present with >90% homology with UniProt chicken proteins and of them, around 70% were present with high similarity between the predicted and UniProt annotated proteins, demonstrating a good agreement between real and predicted protein sequences derived from our assembly process (> 98%, **Figure 4A**). With this schema, we mapped missing paralogs in NCBI galGal6 genome by selecting proteins between 90-100% identity with *Gallus gallus* UniProt proteins. Since we have the genomic positions of the transcripts that encode for all these proteins, if two proteins have near 100% identity by blast analysis and the correspondent transcripts map within a 500 kb window, these proteins are considered as paralog candidates. Conversely, if the transcript maps to different loci positions, we considered them as homologs [45, 46]. Also, we examined if the transcripts that were associated with novel proteins overlapped with loci of previously annotated genes that were missing in the current annotation due to lack of evidence in the NCBI database. If that is the case, we considered these proteins as isoforms from missing genes.

Additionally, we benchmarked our blast results with the use of eggNOG-mapper, a method employing sequence homology search on a metagenomic scale [47, 48]. The output from eggNOG-mapper was intersected with the previous BLASTp results as described here: https://github.com/cfarkas/annotate_my_genomes/wiki#5-annotate-and-identify-homologs-in-novel-proteins-from-transcriptome. With both methods, we confirmed a substantial amount of novel coding genes encoding for endogenous retrovirus genes (ERVs) and genes containing

homology with Ribonuclease H domains, zinc finger domains (CCHC domain-containing proteins), and olfactory genes, among others (**Figure 4B**, **Supplementary Table 3**). Among the latter, we mapped fifteen missing candidate genes with different unfinished annotation status in NCBI database, including six novel homologous genes and novel paralog genes in chicken, respectively. Of the missing genes, Ubiquitin Specific Peptidase 53 (USP53) was absent in NCBI annotation but was present in the Ensembl annotation whereas Aminoadipate-Semialdehyde Dehydrogenase (AASDH) was missing in both databases (**Figure 4C**, upper and lower, respectively). We also confirmed the existence of an alpha macroglobulin paralog, downstream to A2ML3 loci, located in chromosome 1 of galGal6 genome (**Figure 4D**) and a novel homolog of VCP gene, spanning half of an unmapped contig belonging to chromosome Z (chrZ_NW_020109829v1_random, **Figure 4E**). Since the VCP gene maps to chromosome Z, this novel homolog could be classified as a VCP paralog due to its close blast homology with VCP proteins, but the proximity of this unplaced contig cannot be determined with respect to the VCP gene. (**Supplementary Table 3**).

In summary, our ortholog/paralog assignations of non-annotated coding genes can help to increase annotation of important missing genes and aid to reconcile current annotations instead of choosing a single annotation tool from either the NCBI, Ensembl, and/or other sources, a common practice in next-generation sequencing analysis [49]. Also, these procedures can aid to identify novel ERVs, possibly encoding for functional proteins due to their evolutionary conservation in vertebrates [50].

Discussion

Here, we have developed and presented a hybrid RNA-seq annotation pipeline that helps to increase genomic annotation and allows researchers to discover missing/homologous

genes by integrating previous genomic annotations in various animal genomes, providing a reconciled annotation in GTF format. This pipeline can be used for any organism that has an assembled genome and an NCBI available annotation in GTF format and relies on the use of StringTie as a transcript assembler. By using previously well-known tools, this pipeline can efficiently identify non annotated genes versus reconciled genes and distinguish between coding and non-coding genes. We benchmarked our pipeline against well-known bioinformatic genome annotation pipelines such as BRAKER1/2, TSEBRA and SQANTI3 across transcriptomes from five different species. According to the F1-score, our method performed equal or better than existing pipelines in terms of assembly quality. Moreover, F1-scores using genome-guided GTF assemblies as input for our pipeline were scored with the highest F1 values. Therefore, if curated genome annotations are present for a given genome, it is beneficial to run our pipeline, since our method reconcile the current gene annotation and include novel loci. As proof of a concept, we fully assembled the chicken SSPO gene consisting of 106 exons rather than the previously published 105 exons, demonstrating good functionality in well-annotated genomes such as the chicken genome. Our pipeline assembled five transcripts with coding protein potential derived from the SSPO locus in the SCO. The assembly contained > 100 exon isoforms that are consistent with the presence of high molecular weight bands in the SCO previously reported by western blot using anti SCO-spondin (350-300 kDa) as well as lower bands ranging from 200 to 50 kD, probably corresponding to these smaller isoforms [28]. At the time of writing of this manuscript, the protein sequence of chicken SSPO was recently updated in NCBI (NCBI Reference Sequence: NM_001006351.3, October 25, 2021), the sequence associated with the novel chicken genome assembly bGalGal1.mat.broiler.GRCg7b (assembly accession: GCF_016699485.2). The SSPO protein from bGalGal1 assembly contains the novel assembled *exon 1* described in this manuscript, which was only detected

with annotate_my_genomes method. Therefore, this independent result supports the quality of the SSPO transcripts assembled with our pipeline.

Since genome assemblies often update, this tool can aid in rapidly assigning genomic coordinates to missing genes, by inputting the updated genome assembly and correspondent annotation in the pipeline. This was the case of USP53 and AASDH genes, the latter was missing in all genomic annotations since the galGal4 chicken genome assembly was released in 2004 [51]. Also, we discovered a novel VCP homolog spanning half of an unmapped contig belonging to chromosome Z. We thus encourage researchers in the transcriptomics field to consider performing our novel assembly and re-annotation of RNA-seq data rather than using a single GTF annotation file in their studies. Importantly, in SCO organ development we discovered a myriad of divergent lncRNAs according to *FEELnc* lncRNA classification tool, potentially important in the differentiation of neural stem cells [52]. Overall, we propose that this pipeline will be a useful resource for obtaining a comprehensive view of the transcriptional landscape in each study and will help researchers to characterize novel transcriptomes and increase current genome annotations.

Potential Implications

The present work will have two major impacts on the research community. On one side, our pipeline will facilitate the transcriptomic annotation of hybrid sequencing for research without advanced coding skills. This pipeline is implemented as an easy-to-use package that integrates gold standard methods associated with transcriptome annotation. On the other side, our work advances our understanding of the chicken brain transcriptome by displaying an updated annotation, which includes full-length transcripts with challenging structures to assemble. We expect that our method will be useful for biologists interested in improving transcriptome annotation on a wide range of species, tissue and research areas. As well, our

dataset will help to understand the development of specific brain structures providing a transcriptomic resource that can be consulted by all the community.

Methods

RNA isolation and qPCR

We dissected and pooled SCOs from outbred Gallus gallus embryos at Hamburger-Hamilton (HH) stages HH23 and/or HH30 in cold Phosphate Buffered Saline (PBS) solution. Total RNA was isolated using the RNeasy Mini Kit (QIAGEN). The concentration and quality of RNA were measured using Qubit™ RNA HS Assay Kit (Catalog number: Q32852). For qPCR reactions, we reverse transcribed up to 2 µg of RNA with M-MLV reverse transcriptase (PROMEGA) using 0.25 µg of Anchored Oligo(dT)20 Primer (Invitrogen, Catalog number: 12577011). All assayed primers in qPCR reactions are depicted in **Supplementary Table 4**. We performed qPCR reactions using KAPA SYBR FAST qPCR Master Mix (2X) Kit (Kapa Biosciences) with primer concentrations of 0.4 µM. For all PCR reactions, we used as cycling conditions an initial denaturation at 95 °C for 3 min, then 40 cycles with 95 °C for 5 s for denaturation and 60 °C for 20s of annealing/extension. The melting curve indicates no amplification of unspecific products.

RNA sequencing

We assessed the integrity of five RNA samples from SCO HH23(n=2) and HH30, each one derived from at least 25 pooled animals coming from three different egg layings (n=3), by capillar electrophoresis (Agilent 2100 Bioanalyzer), obtaining RIN values between 8.8-9.5 per sample. Four PacBio RSII Isoform libraries were constructed by using 2 µg of total RNA from HH23 (n=2) and HH30 (n=2) SCO (Cold Spring Harbor Laboratory, Genomic Platform, USA).

Sequencing was performed by using IsoSeq protocol (Pacific Biosciences) with long (>4kb) and standard library enrichment sizes per stage. TruSeq Illumina libraries were prepared (two replicates by sample) and sequenced on a NextSeq Paired-End 150 bp middle output (Cold Spring Harbor Laboratory, Genomic Platform, USA). TruSeq Illumina libraries were prepared (two replicates by sample) and sequenced on a NextSeq Paired-End 150 bp middle output (Cold Spring Harbor Laboratory, Genomic Platform, USA).

Isoform Assembly and gene annotation with annotate_my_genomes pipeline.

We aligned PacBio reads against Gallus gallus genome (galGal6 version, GenBank assembly accession GCA_000002315.5) using *minimap2* aligner [30] obtaining depths of ~ 34x. Illumina reads were automatically trimmed using fastp tool [31] and aligned against the referred Gallus gallus genome using HISAT2 aligner [32], obtaining depths of ~ 10x. We sorted, assessed depth, and indexed bam files with *SAMtools* [53]. We merged the resulting BAM files from PacBio and Illumina read alignments into a single BAM file and we assembled transcripts from the latter alignment file using *StringTie* [54, 55] program with settings: -p 1 -j 2 -c 2 -v -a 4. We input assembled transcripts in GTF format to *annotate_my_genomes* pipeline (https://github.com/cfarkas/annotate_my_genomes), obtaining coding/noncoding annotations and reconciled GTF file with current UCSC/NCBI genome annotation references. To reconcile transcripts and correspondent genes with reference genome annotations, the pipeline involves the use of standard UNIX tools, BEDtools [56], and GFF utilities [44] for GTF/GFF3 manipulations. First, we obtained transcripts from the input GTF file using *gffread* [44], and we used the GAWN pipeline (https://github.com/enormandeau/gawn) to initially annotate all possible proteins from the resulting transcripts using Blastx [57] and the Swissprot database, setting the following parameters: -evalue 1e-5 -qcov_hsp_perc 10 [58]. In these steps, the chicken genome (galGal6 genome) was indexed using the *GMAP aligner* [59]. Then, we

assessed long noncoding RNAs training *FEELnc* classifier [34] with known coding RNAs from chicken ("NM_" prefix transcripts). Once lncRNAs were classified, the remaining unclassified transcripts were assessed to predicted coding regions and deduced proteins using *TransDecoder* gene prediction program (https://github.com/TransDecoder/TransDecoder). In this setting, we obtained coding genes, long-noncoding RNAs, and other genes (not classified as coding nor long-noncoding) merged in a single GTF file, indicating known genes by its USCS/NCBI symbol and novel genes with a "STRG" prefix. The installation of annotate_my_genomes pipeline can be achieved through the Conda package manager (https://conda.io) [60], and via Nextflow workflow framework (https://www.nextflow.io/) [61], which allow running the package as a self-contained pipeline.

We visualized BAM files including annotated GTF files with IGV viewer [62]. We plotted GTF files from Figure 3 using standalone pyGenomeTracks python package, available here: https://github.com/deeptools/pyGenomeTracks [63]. In order to calculate the Ex90N50 metric, we used a custom script that uses Salmon program [64]. Finally, we used *BUSCO tool* [65] to assess transcriptome completeness of PacBio and Illumina individual or combined assemblies.

Pipeline Benchmarking

We downloaded, installed, and ran BRAKER1, BRAKER2, TSEBRA, AUGUSTUS and SQANTI3 pipelines along with our method using as inputs the following datasets: our *Gallus gallus* (galGal6) long and short read RNA-seq of the subcommissural organ (European Nucleotide Archive accession numbers PRJEB36569 and PRJEB36584, respectively), *Homo sapiens* (hg38) long-read cDNA sequencing of HAP1 cells (NCBI BioProject PRJNA673144), *Mus musculus* (mm10) long read sequencing of preimplantation embryo transcriptome (NCBI BioProject PRJNA577068), *Danio rerio* (danRer11) long-read sequencing transcriptome

during zygotic genome activation (NCBI BioProject PRJNA395690) and *Caenorhabditis elegans* (ce11) nanopore based direct-RNA sequencing across larvae development (European Nucleotide Archive accession number PRJEB31791) datasets, respectively. The dataset acquisition, preprocessing, genomic alignments and pipeline executions are described in detail here: https://github.com/cfarkas/annotate_my_genomes/wiki/annotate_my_genomes-benchmarking. The precision, recall, and their harmonic mean - the F1-score - as measures of gene prediction accuracy were obtained by using gffcompare.

Homolog Assignments

To assess possible homologs in novel coding genes (cds), we blasted the novel predicted proteins from these cds against the UniProt *Gallus gallus* proteome (taxid 9031) [66] with the setting -max_hsps 1 -max_target_seqs 1 in blastp command [67]. Then, we parsed these results and compared the genomic positions of all novel protein matches against the genomic positions of proteome indexed in NCBI. If two matches with 90-100% homology were found within the same loci (<0.5 Mb), we considered them as paralogs [45, 46]. Otherwise, we considered these matches as missing genes in the reference annotation. We also integrated to the previous results the metagenome-level annotation of novel proteins using eggNOG-mapper ortholog classification software [47, 48]. All relevant commands to reproduce these analyses are available here: https://github.com/cfarkas/annotate_my_genomes/wiki#5-annotate-and-identify-homologs-in-novel-proteins-from-transcriptome

Immunohistochemistry

Immunohistochemistry was performed following the protocol described in Vera et al. (2013), using anti-NCAM cytoplasmic domain antibody (4D from Developmental Studies Hybridoma Bank, University of Iowa, Iowa City, IA) as well as with a rabbit anti Reissner's fiber glycoproteins antibody (AFRU) that recognizes SCO-spondin [28]. As second antibodies

we used Goat anti-mouse Alexa-546 and anti-rabbit Alexa-488 antibodies (Invitrogen, Carlsbad, CA), and nuclei were visualized with TOPRO-3 (Invitrogen, Carlsbad, CA).

Data and Code Availability

All computational steps to replicate the analysis performed in this paper are available here: https://github.com/cfarkas/annotate_my_genomes. We provide on the GitHub page an easy-to-install package of our pipeline that can be run on a modern laptop using Linux/Ubuntu operating system. PacBio and Illumina RNA-sequencing datasets are available at European Nucleotide Archive (ENA) Accession Number PRJEB36569 (PacBio) and PRJEB36584 (Illumina).

Availability and requirements:

Project name: annotate_my_genomes (version 2.1, March 2022)

Project home page: https://github.com/cfarkas/annotate_my_genomes

Operating system(s): Ubuntu/MacOSX

Programming language: BASH, Python, NextFlow

Other requirements: ncbi-blast+ version equal or higher than v2.7.1, SAMtools and Python3.

License: MIT License

Figure Legends

Figure 1: Combined PacBio and Illumina RNA sequencing assembly improves gene annotation in the chicken transcriptome. A) Schematic diagram of annotate_my_genomes pipeline. PacBio and Illumina reads are aligned to a given reference genome, then assembled to isoforms by *StringTie*. By combined homology identification and coding/noncoding RNA classification, we annotated the resulting GTF file, identifying previously annotated genes (i.e. USCS/NCBI) and missing genes, respectively (see annotate_my_genomes). **B**) Various transcriptome metrics for StringTie assemblies based solely on Illumina, PacBio (IsoSeq), and merged technologies (see green, red, and black dots, respectively). From left to right, we show Ex90N50, bases, and number of assembled transcripts, respectively. **C**) BUSCO classification of single-copy and multicopy orthologs in each transcriptome assembly. Complete, fragmented, and missing orthologs are depicted with different colors. **D**) (*left*) Number of coding, noncoding, and other assembled transcripts (other features) in reconciled 19,690 genes with NCBI current annotation for galGal6 in June 2020. (*Right*) Same as left for 4,292 non annotated genes in NCBI current annotation for galGal6 in June 2020. **E**) Same as (D) for the number of transcripts. **F**) Classification of 10,912 annotated long noncoding RNAs by location using FEELnc tool. **G**) Classification of lncRNA by Type using FEELnc tool.

Figure 2: Giant SSPO gene is fully reconstructed by hybrid sequencing technology. A) Coverage of PacBio and Illumina alignment at *SSPO* locus (chr2:466581-503024 in galGal6 assembly) at HH23 (magenta) and HH30 (blue) stages, visualized by IGV viewer. The red-colored track indicates the coverage of merged Illumina BAM file including the correspondent assembled isoforms underneath in blue. Similarly, we included as a red-colored track the

coverage of merged PacBio and Illumina sequencing including the assembled isoforms underneath in blue. We highlighted in blue numbers the beginning and last exons of assembled *SSPO* isoforms and in green numbers the alternative isoform usage across *SSPO* isoforms. We included the current galGal6a annotation underneath all tracks in blue color. **B**) (*left*) PCR product of three sets of primers spanning SSPO gene at exons 8-9 (setA), 23-24 (setB) and 105-106 (setC), from RNA of SCO at stage HH30. (*right*) qPCR of the three referred primer sets from SCO RNA derived from at least ten pooled animals coming from four different egg laying at stage HH30. Significance of comparisons was assessed with Student's t test (P<0.05 *, P<0.01 **, P<0.001 ***, P>0.05 ns) **C**) Immunohistochemistry of SCO-spondin and NCAM in the SCO at HH23 (upper) and HH30 (lower) stages. TOPRO3 in blue, NCAM in red, and SCO-spondin in green, Di: Diencephalon, Mes: Mesencephalon. Significance of comparisons were assessed with Student's t test (P<0.05 *, P<0.01 **, P<0.001 ***, P>0.05 ns).

Figure 3**: Pipeline benchmarking.** A) Coverage of PacBio and Illumina alignment at SSPO locus (chr2:466581-503024 in galGal6 assembly) at HH23 (red) and HH30 (purple) stages, plotted with pyGenomeTracks, including SSPO gene models resolved with annotate_my_genomes (genome-guided, green tracks), BRAKER2 (yellow tracks), TSEBRA (brown tracks), AUGUSTUS (grey tracks), SQANTI3 (no plotted tracks at this position) and galGal6_ncbiRefSeq (dark blue track), respectively. The latter track corresponds to the reference track provided by NCBI. The light blue-colored track indicates a novel assembled exon corresponding to the first exon of SSPO (exon 1). **B)** Detailed view of the first assembled exon by annotate_my_genomes, Light-blue annotations indicate exon numbers. **C)** Heatmap depicting F1-score calculations comparing the output GTF from each method against the NCBI annotations (galGal6_ncbiRefSeq.gtf) on the base, exon, intron, intron-chain, transcript, and locus level parameters, respectively. All employed methods are enlisted on the left of the graph.

The F1 score was calculated based on precision and recall values obtained on each parameter from gffcompare. Black asterisks indicate the best F1 scores per method. The color scale indicates lower and higher F1 values with blue and yellow scales, respectively. **D)** Same as C) for Mus musculus (mm10), Homo sapiens (hg38), Danio rerio (danRer11) and Caenorhabditis elegans (ce11) genome RNA-seq datasets, respectively. The benchmarked methods were the following: annotate_my_genomes (with or without genome guide) BRAKER2, TSEBRA, AUGUSTUS *Ab initio,* and SQANTI3. Black asterisks indicate the best F1 scores per method. The color scale indicates lower and higher F1 values with blue and yellow scales, respectively.

Figure 4: Homolog and paralog assignments successfully mapped missing genes in chicken reference annotations. A) BLASTp identities (in percentage) obtained by blasting the deduced proteome from the assembled transcriptome against all *Gallus gallus* proteome consisting in 34730 uniprot chicken proteins (taxid 9031, https://www.uniprot.org/taxonomy/9031). Colors depict the BLAST identity percentage. **B**) Pie plot of 163 missing proteins displaying 90-100% identity with UniProt proteins. **C-E**) Missing genes in galGal6 reference, discovered by our pipeline. We denoted in green the candidate gene coverage including the assembled transcripts while we denoted in red the coverage of neighbor genes. (C) USP53 paralog, **D**) A2M paralog in chicken genome assigned to chromosome 1. **E**) Novel VCP homolog discovered in an unplaced contig belonging to chromosome Z.

Additional Files

Supplementary Figure 1: Illumina sequencing coverage at *SSPO* locus (chr2:466581-503024 in galGal6 assembly) of whole-brain RNA extracted at stages HH31 and HH36, respectively

(see tracks with green names). Illumina sequencing coverage at *SSPO* locus (chr2:466581-503024 in galGal6 assembly) of SCO RNA extracted at stages HH23 and HH30, respectively (see tracks with blue names). Tracks were obtained from galGal6 UCSC genome browser (https://genome.ucsc.edu/).

Supplementary Figure 2: **A**) Coverage plots of all assembled SSPO isoforms found with the pipeline, obtained with RSEQC. Each line color denotes the coverage of each Illumina sequencing across the gene body percentiles (V41.sorted = HH23_rep1, V42.sorted = HH23_rep2, V71.sorted = HH30_rep1 and V72.sorted = HH30_rep2). **B)** Same as A) just considering 5’ and 3’ end SSPO isoforms (transcripts N° STRG.7690.2 and STRG.7690.5, see Fig 2).

Supplementary Figure 3: CPU time executions of the different assayed methods across RNA-seq datasets employed in this study. AmyG = annotate_my_genomes.

Supplementary Table 1: Annotation of the assembled transcriptome with hybrid sequencing technologies, including predicted long-noncoding RNAs. Reference Transcript annotation sheet contains StringTie transcripts intersections with NCBI reference transcripts, including correspondent transcript sequences (n=61679). Novel Transcript Annotation sheet contains novel transcripts including correspondent transcript sequences (n=5610). Candidate lncRNA classes contain all transcripts classified as lncRNAs by *FEELnc* software.

Supplementary Table 2: F1-scores, calculated with precision and recall values from gffcompare across RNA-seq datasets employed in this study. Precision and recall values were obtained by comparing base, exon, intron, intron-chain, transcript, and locus level between

output GTF from several genome annotation pipelines, and the reference GTF annotation from NCBI (considered as truth).

Supplementary Table 3: Ortholog and paralog identification within novel *Gallus gallus* proteins. **Sheet 1** contains eggNOG-mapper ortholog annotations from novel proteins. **Sheet 2** contains blastp results from novel proteins against Gallus Gallus uniprot database (taxid 9031). The latter results were filtered with a cut-off between 90-100% identity. **Sheet 3** contains the intersection between the refereed datasets. **Sheet 4** contains manual annotation of the latter intersection, respectively.

Supplementary Table 4: List of publicly available sequencing datasets and primers used in this study.

Abbreviations

AASDH: Aminoadipate-Semialdehyde Dehydrogenase
CCS: circular consensus sequences
ERVs: Endogenous retrovirus genes
GTF: Gene Transfer Format
LncRNA: long non-coding RNAs
NGS: Next Generation Sequencing
ON: Oxford Nanopore
RNA-seq: RNA-sequencing
SCO: sub-commissural organ
SMRT: single-molecule real-time
SSPO: SCO-spondin:
USP53: Ubiquitin Specific Peptidase 53
VCP: Transitional endoplasmic reticulum ATPase
HH: Hamburger-Hamilton

Competing interests

The authors declare that they have no competing interests

Funding

This work was supported by Fondo Nacional de Desarrollo Científico y Tecnológico, FONDECYT [1191860 to T.C] and FONDECYT de Iniciación [11190401 to ETS]. CF and JJH received partial funding from the CIHR and CancerCare Manitoba Foundation.

Authors' Contributions

TC and CF: conceived and designed the experiments. CF, AR: were involved in sample preparation, qPCR, and IHC. CF, ETS, and CDH: were involved in data analysis and figure processing. CF, AM and MGO implemented the pipeline in both Anaconda and NextFlow environments. CF, TC, JJH, ETS: were involved in results discussion and paper writing.

Ethical Statement

The ethics, bioethics and biosafety committee of Vicerrectoría de Investigación y Desarrollo de la Universidad de Concepción has been reviewed the proposal of the project "PROYECTO Nº 1191860", adjudicated from CONCURSO FONDECYT REGULAR 2019, entitled "SCO-SPONDIN: A CEREBROSPINAL FLUID MATRICELLULAR PROTEIN FULFILLING CRUCIAL NEUROGENIC FUNCTIONS" proposed by the principal investigator DRA. TERESA CAPRILE ELOLA-OLASO, ascribed professor at Departamento de Biología Celular de la Facultad de Ciencias Biológicas de la Universidad de Concepción, has confirmed that fulfill the national and international established norms, ethical and bioethical principles and biosafety procedures regarding animal handling and experimentation (in this case, Gallus gallus embyos), including the appropriate handling of chemical biological waste derived from this proposal. The approval of this document is follows gudelines of

National law Res. Exenta Nº 157, del 24 de enero de 2013 from CONICYT. Reference document: CEBB 408-2019.

Acknowledgments

Powered@NLHPC: This research was partially supported by the supercomputing infrastructure of the NLHPC (ECM-02) and supported by the high-performance computing system of PIDi-UTEM (SCC-PIDi-UTEM CONICYT - FONDEQUIP - EQM180180).

References

1. Ellegren, H., *Genome sequencing and population genomics in non-model organisms.* Trends Ecol Evol, 2014. **29**(1): p. 51-63.
2. Sahraeian, S.M.E., et al., *Gaining comprehensive biological insight into the transcriptome by performing a broad-spectrum RNA-seq analysis.* Nat Commun, 2017. **8**(1): p. 59.
3. Pop, M., *Genome assembly reborn: recent computational challenges.* Brief Bioinform, 2009. **10**(4): p. 354-66.
4. Alkan, C., S. Sajjadian, and E.E. Eichler, *Limitations of next-generation genome sequence assembly.* Nat Methods, 2011. **8**(1): p. 61-5.
5. Jiao, W.B., et al., *Improving and correcting the contiguity of long-read genome assemblies of three plant species using optical mapping and chromosome conformation capture data.* Genome Res, 2017. **27**(5): p. 778-786.
6. Amarasinghe, S.L., et al., *Opportunities and challenges in long-read sequencing data analysis.* Genome Biol, 2020. **21**(1): p. 30.
7. Wenger, A.M., et al., *Accurate circular consensus long-read sequencing improves variant detection and assembly of a human genome.* Nat Biotechnol, 2019. **37**(10): p. 1155-1162.
8. Zhang, S.J., et al., *Isoform Evolution in Primates through Independent Combination of Alternative RNA Processing Events.* Mol Biol Evol, 2017. **34**(10): p. 2453-2468.
9. Liu, X., et al., *Detecting alternatively spliced transcript isoforms from single-molecule long-read sequences without a reference genome.* Mol Ecol Resour, 2017. **17**(6): p. 1243-1256.
10. Sahlin, K., et al., *Deciphering highly similar multigene family transcripts from Iso-Seq data with IsoCon.* Nat Commun, 2018. **9**(1): p. 4601.
11. Prjibelski, A.D., et al., *Extending rnaSPAdes functionality for hybrid transcriptome assembly.* BMC Bioinformatics, 2020. **21**(Suppl 12): p. 302.

12. Puglia, G.D., et al., *Hybrid transcriptome sequencing approach improved assembly and gene annotation in Cynara cardunculus (L.).* BMC Genomics, 2020. **21**(1): p. 317.
13. Fu, S., et al., *IDP-denovo: de novo transcriptome assembly and isoform annotation by hybrid sequencing.* Bioinformatics, 2018. **34**(13): p. 2168-2176.
14. Bushmanova, E., et al., *rnaQUAST: a quality assessment tool for de novo transcriptome assemblies.* Bioinformatics, 2016. **32**(14): p. 2210-2.
15. Tardaguila, M., et al., *SQANTI: extensive characterization of long-read transcript sequences for quality control in full-length transcriptome identification and quantification.* Genome Res, 2018.
16. Venturini, L., et al., *Leveraging multiple transcriptome assembly methods for improved gene structure annotation.* Gigascience, 2018. **7**(8).
17. Holt, C. and M. Yandell, *MAKER2: an annotation pipeline and genome-database management tool for second-generation genome projects.* BMC Bioinformatics, 2011. **12**: p. 491.
18. Cantarel, B.L., et al., *MAKER: an easy-to-use annotation pipeline designed for emerging model organism genomes.* Genome Res, 2008. **18**(1): p. 188-96.
19. Hoff, K.J. and M. Stanke, *WebAUGUSTUS--a web service for training AUGUSTUS and predicting genes in eukaryotes.* Nucleic Acids Res, 2013. **41**(Web Server issue): p. W123-8.
20. Stanke, M., et al., *AUGUSTUS: ab initio prediction of alternative transcripts.* Nucleic Acids Res, 2006. **34**(Web Server issue): p. W435-9.
21. Hoff, K.J., et al., *BRAKER1: Unsupervised RNA-Seq-Based Genome Annotation with GeneMark-ET and AUGUSTUS.* Bioinformatics, 2016. **32**(5): p. 767-9.
22. Lukashin, A.V. and M. Borodovsky, *GeneMark.hmm: new solutions for gene finding.* Nucleic Acids Res, 1998. **26**(4): p. 1107-15.
23. Bruna, T., et al., *BRAKER2: automatic eukaryotic genome annotation with GeneMark-EP+ and AUGUSTUS supported by a protein database.* NAR Genom Bioinform, 2021. **3**(1): p. lqaa108.
24. Gabriel, L., et al., *TSEBRA: transcript selector for BRAKER.* BMC Bioinformatics, 2021. **22**(1): p. 566.
25. Cook, D.E., et al., *Long-Read Annotation: Automated Eukaryotic Genome Annotation Based on Long-Read cDNA Sequencing.* Plant Physiol, 2019. **179**(1): p. 38-54.
26. Treutlein, B., et al., *Cartography of neurexin alternative splicing mapped by single-molecule long-read mRNA sequencing.* Proc Natl Acad Sci U S A, 2014. **111**(13): p. E1291-9.
27. Anvar, S.Y., et al., *Full-length mRNA sequencing uncovers a widespread coupling between transcription initiation and mRNA processing.* Genome Biol, 2018. **19**(1): p. 46.
28. Vera, A., et al., *SCO-spondin from embryonic cerebrospinal fluid is required for neurogenesis during early brain development.* Front Cell Neurosci, 2013. **7**: p. 80.
29. Cantaut-Belarif, Y., et al., *The Reissner Fiber in the Cerebrospinal Fluid Controls Morphogenesis of the Body Axis.* Curr Biol, 2018. **28**(15): p. 2479-2486 e4.
30. Li, H., *Minimap2: pairwise alignment for nucleotide sequences.* Bioinformatics, 2018. **34**(18): p. 3094-3100.
31. Chen, S., et al., *fastp: an ultra-fast all-in-one FASTQ preprocessor.* Bioinformatics, 2018. **34**(17): p. i884-i890.

32. Kim, D., et al., *Graph-based genome alignment and genotyping with HISAT2 and HISAT-genotype.* Nat Biotechnol, 2019. **37**(8): p. 907-915.
33. Altschul, S.F., et al., *Gapped BLAST and PSI-BLAST: a new generation of protein database search programs.* Nucleic Acids Res, 1997. **25**(17): p. 3389-402.
34. Wucher, V., et al., *FEELnc: a tool for long non-coding RNA annotation and its application to the dog transcriptome.* Nucleic Acids Res, 2017. **45**(8): p. e57.
35. Dimmer, E.C., et al., *The UniProt-GO Annotation database in 2011.* Nucleic Acids Res, 2012. **40**(Database issue): p. D565-70.
36. Reuter, J.A., D.V. Spacek, and M.P. Snyder, *High-throughput sequencing technologies.* Mol Cell, 2015. **58**(4): p. 586-97.
37. Palazzo, A.F. and E.S. Lee, *Non-coding RNA: what is functional and what is junk?* Front Genet, 2015. **6**: p. 2.
38. Warnecke, T. and L.D. Hurst, *Error prevention and mitigation as forces in the evolution of genes and genomes.* Nat Rev Genet, 2011. **12**(12): p. 875-81.
39. Djebali, S., et al., *Landscape of transcription in human cells.* Nature, 2012. **489**(7414): p. 101-8.
40. Gobron, S., et al., *Subcommissural organ/Reissner's fiber complex: characterization of SCO-spondin, a glycoprotein with potent activity on neurite outgrowth.* Glia, 2000. **32**(2): p. 177-91.
41. Meiniel, A., *SCO-spondin, a glycoprotein of the subcommissural organ/Reissner's fiber complex: evidence of a potent activity on neuronal development in primary cell cultures.* Microsc Res Tech, 2001. **52**(5): p. 484-95.
42. Didier, R., O. Meiniel, and A. Meiniel, *Molecular cloning and early expression of chick embryo SCO-spondin.* Cell Tissue Res, 2007. **327**(1): p. 111-9.
43. Wang, L., S. Wang, and W. Li, *RSeQC: quality control of RNA-seq experiments.* Bioinformatics, 2012. **28**(16): p. 2184-5.
44. Pertea, G. and M. Pertea, *GFF Utilities: GffRead and GffCompare.* F1000Res, 2020. **9**.
45. Parrish, P.C.R., et al., *Discovery of synthetic lethal and tumor suppressor paralog pairs in the human genome.* Cell Rep, 2021. **36**(9): p. 109597.
46. Yokomine, T., et al., *Structural and functional analysis of a 0.5-Mb chicken region orthologous to the imprinted mammalian Ascl2/Mash2-Igf2-H19 region.* Genome Res, 2005. **15**(1): p. 154-65.
47. Cantalapiedra, C.P., et al., *eggNOG-mapper v2: Functional Annotation, Orthology Assignments, and Domain Prediction at the Metagenomic Scale.* Mol Biol Evol, 2021. **38**(12): p. 5825-5829.
48. Huerta-Cepas, J., et al., *eggNOG 5.0: a hierarchical, functionally and phylogenetically annotated orthology resource based on 5090 organisms and 2502 viruses.* Nucleic Acids Res, 2019. **47**(D1): p. D309-D314.
49. Zhao, S. and B. Zhang, *A comprehensive evaluation of ensembl, RefSeq, and UCSC annotations in the context of RNA-seq read mapping and gene quantification.* BMC Genomics, 2015. **16**: p. 97.
50. Johnson, W.E., *Origins and evolutionary consequences of ancient endogenous retroviruses.* Nat Rev Microbiol, 2019. **17**(6): p. 355-370.
51. International Chicken Genome Sequencing, C., *Sequence and comparative analysis of the chicken genome provide unique perspectives on vertebrate evolution.* Nature, 2004. **432**(7018): p. 695-716.

52. Luo, S., et al., *Divergent lncRNAs Regulate Gene Expression and Lineage Differentiation in Pluripotent Cells.* Cell Stem Cell, 2016. **18**(5): p. 637-52.
53. Li, H., et al., *The Sequence Alignment/Map format and SAMtools.* Bioinformatics, 2009. **25**(16): p. 2078-9.
54. Pertea, M., et al., *StringTie enables improved reconstruction of a transcriptome from RNA-seq reads.* Nat Biotechnol, 2015. **33**(3): p. 290-5.
55. Pertea, M., et al., *Transcript-level expression analysis of RNA-seq experiments with HISAT, StringTie and Ballgown.* Nat Protoc, 2016. **11**(9): p. 1650-67.
56. Quinlan, A.R., *BEDTools: The Swiss-Army Tool for Genome Feature Analysis.* Curr Protoc Bioinformatics, 2014. **47**: p. 11 12 1-34.
57. Altschul, S.F., et al., *Basic local alignment search tool.* J Mol Biol, 1990. **215**(3): p. 403-10.
58. Bairoch, A. and R. Apweiler, *The SWISS-PROT protein sequence database and its supplement TrEMBL in 2000.* Nucleic Acids Res, 2000. **28**(1): p. 45-8.
59. Wu, T.D. and C.K. Watanabe, *GMAP: a genomic mapping and alignment program for mRNA and EST sequences.* Bioinformatics, 2005. **21**(9): p. 1859-75.
60. Gruning, B., et al., *Bioconda: sustainable and comprehensive software distribution for the life sciences.* Nat Methods, 2018. **15**(7): p. 475-476.
61. Di Tommaso, P., et al., *Nextflow enables reproducible computational workflows.* Nat Biotechnol, 2017. **35**(4): p. 316-319.
62. Thorvaldsdottir, H., J.T. Robinson, and J.P. Mesirov, *Integrative Genomics Viewer (IGV): high-performance genomics data visualization and exploration.* Brief Bioinform, 2013. **14**(2): p. 178-92.
63. Ramirez, F., et al., *High-resolution TADs reveal DNA sequences underlying genome organization in flies.* Nat Commun, 2018. **9**(1): p. 189.
64. Patro, R., et al., *Salmon provides fast and bias-aware quantification of transcript expression.* Nat Methods, 2017. **14**(4): p. 417-419.
65. Simao, F.A., et al., *BUSCO: assessing genome assembly and annotation completeness with single-copy orthologs.* Bioinformatics, 2015. **31**(19): p. 3210-2.
66. UniProt, C., *UniProt: the universal protein knowledgebase in 2021.* Nucleic Acids Res, 2021. **49**(D1): p. D480-D489.
67. Healy, M.D., *Using BLAST for performing sequence alignment.* Curr Protoc Hum Genet, 2007. **Chapter 6**: p. Unit 6 8.

Figure 1

Click here to access/download;Figure;Fig1.pdf

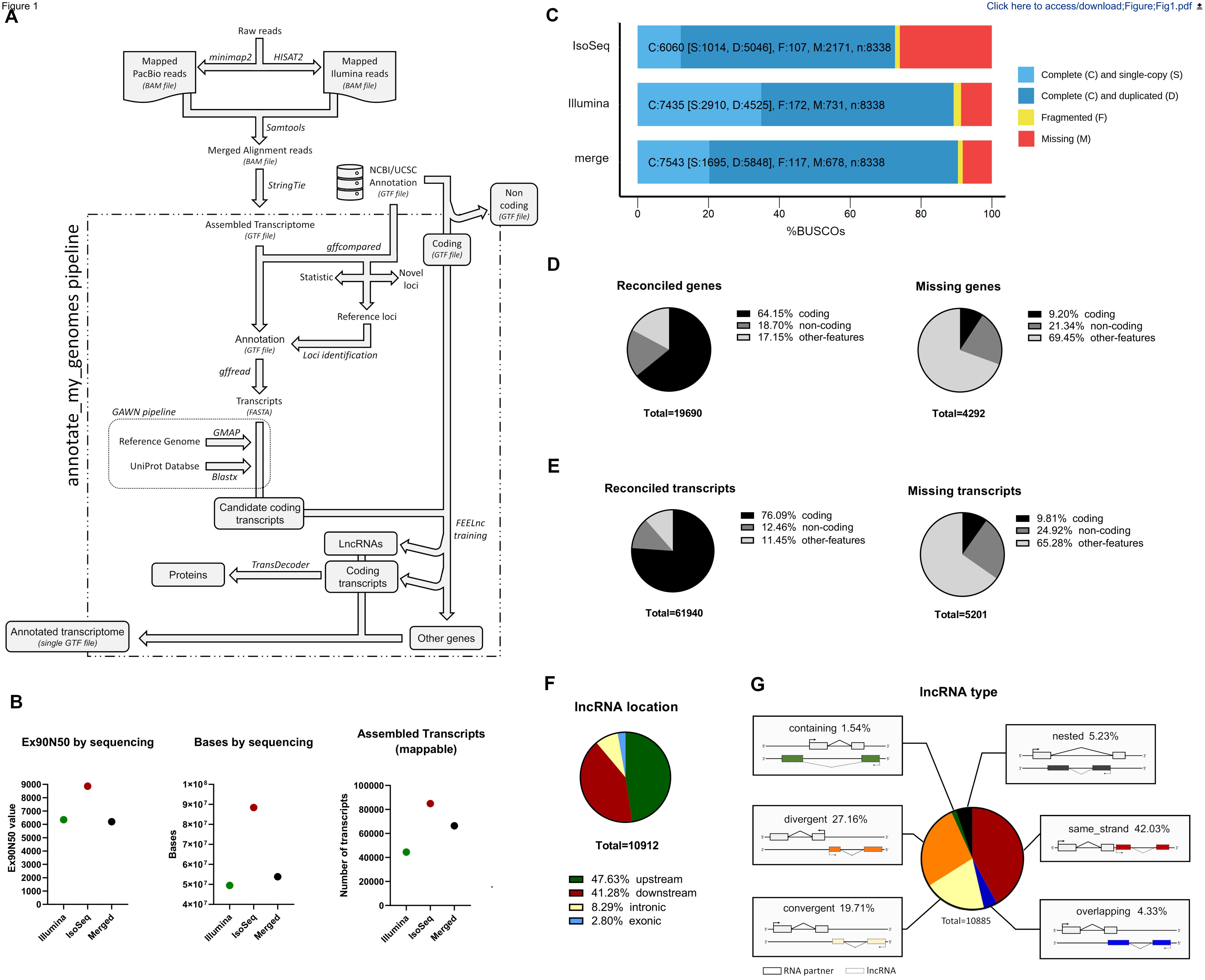

Figure 2

Click here to access/download;Figure;Fig2.pdf

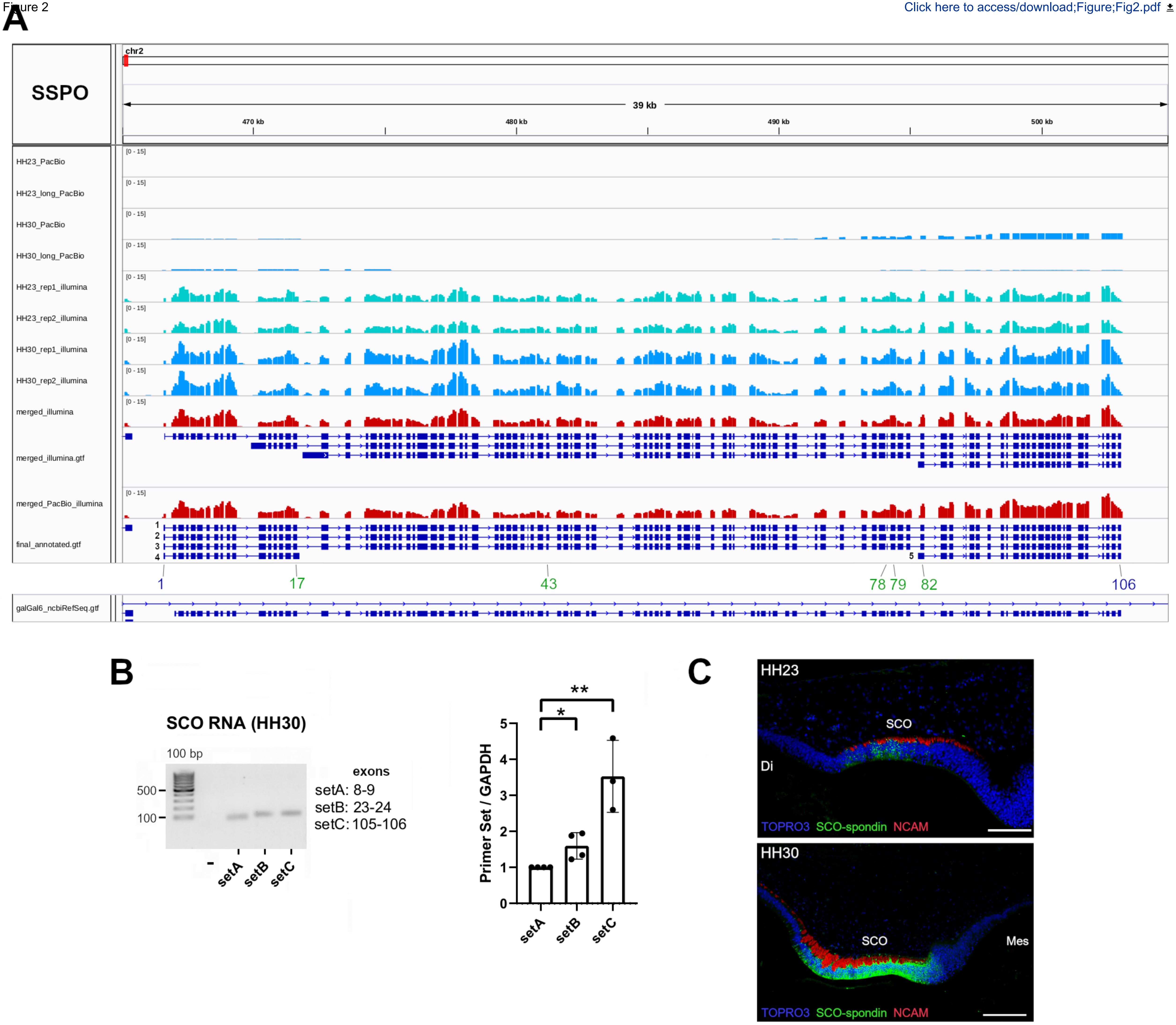

Figure 3

Click here to access/download;Figure;Fig3.pdf

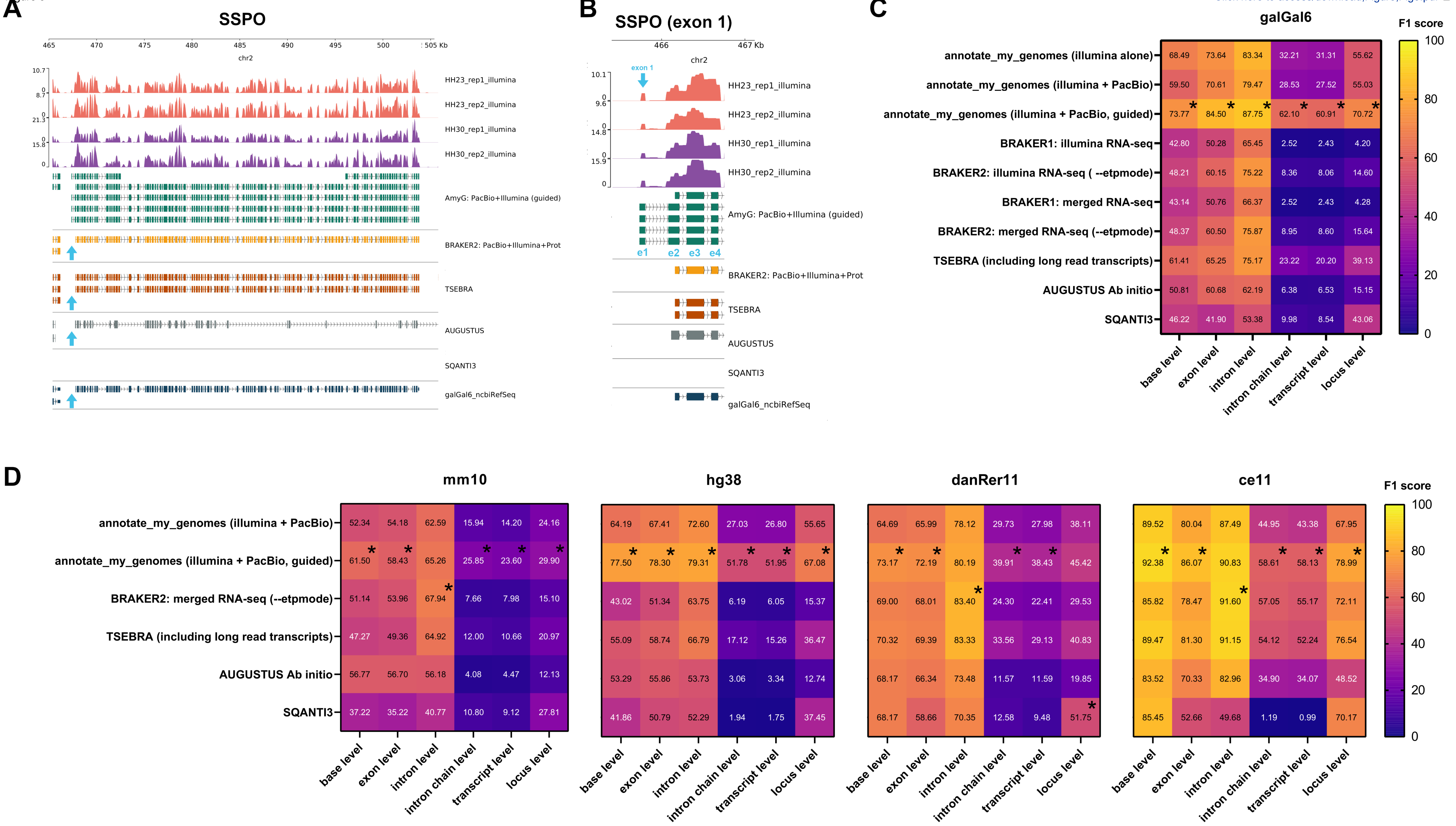

Click here to access/download;Figure;Fig4.pdf

Figure 4

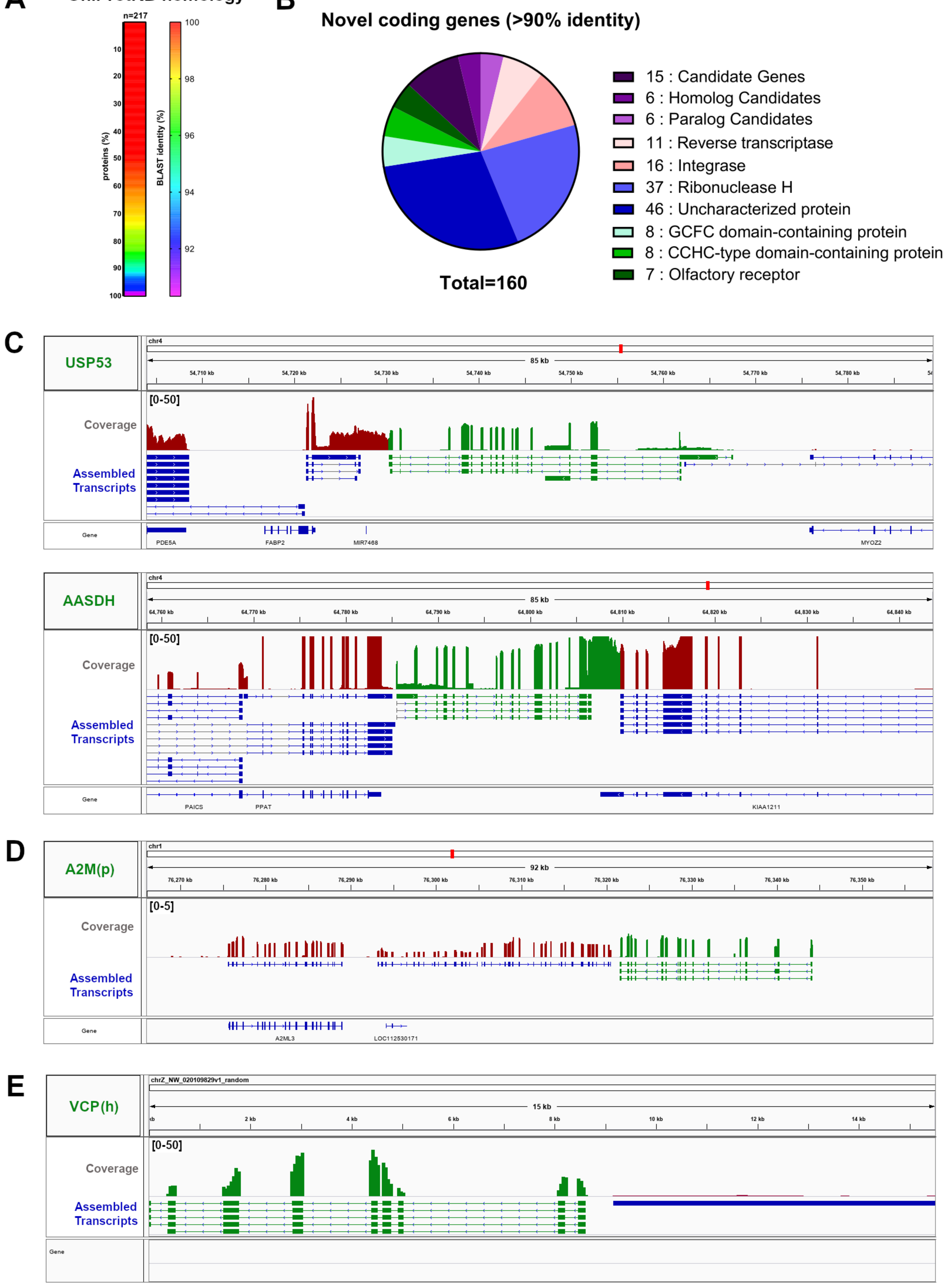

Supplementary Figure 1

Click here to access/download
Supplementary Material
Supplementary_Figure_1.pdf

Supplementary Figure 2

Click here to access/download
Supplementary Material
Supplementary_Figure_2.pdf

Supplementary Figure 3

Click here to access/download
Supplementary Material
Supplementary_Figure_3.pdf

Supplementary Table 1

Click here to access/download
Supplementary Material
Supp1-Annotation.xlsx

Supplementary Table 2

Click here to access/download
Supplementary Material
Supp2-F1_score.xlsx

Supplementary Table 3

Click here to access/download

Supplementary Material

Supp3-paralog_candidates.xlsx

Supplementary Table 4

Click here to access/download
Supplementary Material
Supp4-Sequencing_datasets_and_qPCR_primers.xlsx

Response to reviewers

Click here to access/download
Supplementary Material
Response to Reviewers .pdf

Cover Letter

Click here to access/download;Personal Cover;CoverLetter_GigaScience_22032022.docx

Universidad de Concepción

100 Años UdeC

Research Institute in Oncology and Hematology

University of Manitoba

CancerCare Manitoba

March, 2022

Dr. Nicole Nogoy
Editor GigaScience

Dear Dr. Nicole Nogoy

Please find attached our new version of the manuscript entitled 'A*nnotate_my_genomes: an easy-to-use pipeline to improve genome annotation and uncover neglected genes by hybrid RNA sequencing'* that we would like to be considered for publication in *GigaScience*.

A previous version of this manuscript was sent in August 2021 (GIGA-D-21-00247), but on that occasion, the reviewers considered that it was necessary to improve the pipeline giving us several suggestions on this matter. We appreciate the revision made that largely improved our work. Now, we are sending a new version of our manuscript with all the reviewers' concerns fully addressed. One of the most important improvements we made to our pipeline involves the implementation as an Anaconda environment and as a Nextflow container workflow, providing different alternatives to the users, including reproducibility. On the other hand, we benchmarked our pipeline with different genome annotation workflows such as AUGUSTUS, BRAKER1, BRAKER2, the recent TSEBRA method, and PacBio-dedicated SQANTI3 workflow. Annotate_my_genomes, relying on StringTie assembler, often displayed equal or superior performance based on the F1-score against truth (please see Figure 3).

As stated in the initial submission, our study provides a user-friendly bioinformatic tool that performs genome-guided transcriptome annotation, using as input assembled transcripts from hybrid sequencing such PacBio and Illumina technology. This pipeline distinguishes between coding and long non-coding RNAs by the integration of several bioinformatic approaches, including gene reconciliation with previous annotations in Gene Transfer Format (GTF). Although the use of this pipeline was originally designed to annotate assembled transcripts from hybrid sequencing, it can also be applied to annotate assembled transcripts from mapped data derived from Illumina alone or other sequencing technologies.

We first demonstrated the efficiency of our approach by correctly assembling and annotating all exons from the chicken SCO-spondin gene (containing more than 105 exons), a giant gene that consistently lacks coding information in NCBI or Ensembl databases. This gene encodes a protein of more than 5000 amino acids and its expression is highly conserved in Chordates and restricted to the sub-commissural organ, a brain gland related to different morphogenic events, such as the regulation of brain development and body axis alignment. We also successfully mapped hundreds of missing genes in the chicken reference annotations by homology assignments, including the characterization of USP53 and AASDH genes. As an example, the latter gene is missing in all genomic annotations since the chicken genome assembly was released in 2004 (galGal4). In this sense, our method not only allows us to annotate new transcripts but also reconcile current annotations providing a compressive result.

Below, we will proceed to detail the responses to all the comments and requests made by the reviewers:

Reviewer #1:

The manuscript "annotate_my_genomes: an easy-to-use pipeline to improve genome annotation and uncover neglected genes by hybrid RNA sequencing" by Farkas et. al. present a pipeline to annotate genomes with available genome annotations. The manuscript is well written in a clear and concise way. The authors present a methodology to integrate long-read RNA-Seq data with short-read to generate the assembly. The annotation pipeline uses also a

combination of Illumina (short-reads) and PacBio (long-reads) sequencing.
The authors test the annotation pipeline with chicken brain data. They are able to identify and annotate genes that were missed in the public genome release for this organism.
I consider that there are few issues that should be added to the manuscript.

Q1. StringTie is used as assembler. I think that the manuscript should include a few comments on this step as there are other assembler like Trinity that can be used. There are still discussions about the bias that can be introduced in an assembly if the genome coordinates are used. I would like to know if the authors compared the StringTie non-reference based approach with the reference-based and they opinion about the bias of using annotated genomes files during the assembly.

Ans1. We agreed and thanked the reviewer's suggestion. In our pipeline, we initially used genome-guided mapping (using HISAT2/minimap2), producing a non-reference-based assembly as input for our pipeline (using StringTie). As suggested by this reviewer, we tested reference-based StringTie assemblies, obtaining equal or better F1-scores against truth GTF annotations from NCBI, when compared with non-reference StringTie based assemblies (please see Figure 3). Therefore, we recommend employing reference-based StringTie assemblies in our pipeline if good reference annotations are available. We expressed these observations in the manuscript.
Concerning the use of Trinity, we didn't use this assembler because of their large memory requirements. The module Chrysalis on the Trinity pipeline requires more than 200Gb of RAM when a 28Gb Illumina sequencing dataset is assembled. Last, but not least, Trinity is a de-novo assembler for short reads which doesn't account for long read integration. The commands to create the assembly as we did on the present work are described on our pipeline's Wiki: https://github.com/cfarkas/annotate_my_genomes/wiki#ii-obtaining-stringtie-gtf-file-for-annotation

Q2. The manuscript should include a comparison between the annotation pipeline and the already published pipelines mentioned in the Background section. There should be a discussion on advantages and disadvantages of the author's proposal compared with the existent pipelines.

Ans2. We agreed and thanked the reviewer's suggestion. We have included a new paragraph in the background section (see lines 62-86) mentioning published pipelines. Based on our benchmark, we discussed the advantages and disadvantages (such as sensitivity and precision calls) of the different pipelines (see lines 249-269 in Results section, and lines 329-336 in Discussion section, respectively).

Q3. There should be a discussion about running times and computer resources required by the pipeline. I would like to see, if possible, a time estimation for other organism like human and mouse.

Ans3. We agreed with this reviewer and performed these calculations. A detailed comparison among different pipelines for five organisms is included in Supplementary Figure 3.

Q4. Often, RNA-Seq data contains contamination. How this pipeline can handle contaminated raw reads? If it require non-contaminated reads, then it should be clear in the manuscript

Ans4. We fully agreed with the reviewer. We mention the Illumina preprocessing steps between lines 400 and 405 our pre-processing methodology for Illumina short reads. In this work, we choose the well-known tool fastp, however, it is important to mention that nowadays several tools are available for QC pre-processing; we expect that users choose the one that suits them better according to their dataset.

Q5. I went over the source code. It is mainly complex BATCH scripts. Nowadays, computational biology data analysis pipelines are implemented using workflow languages and executed through workflow managers like CWLtool, Nextflow or snakepipes, just to mention a few. These workflow languages and manager allow portability, scalability and more important reproducibility of the results. The current source code shows a very complex set of

BATCH scripts that, in my personal opinion, are not close to the "easy-to-use" word used in the manuscript title. In my opinion, the authors should analyze the possibility to implement the pipeline using a workflow language. If this is not possible, I would like to know the reasons.

Ans5. We thank the reviewer for such a valuable suggestion. Today, we have implemented our pipeline on the NextFlow workflow and as an Anaconda environment to provide the users with alternatives to run our pipeline. We explicit recommend running our pipeline via NextFlow, since it is easy to install (requires a few lines of code) and ensure reproducibility when implemented in different operative systems (we tested our Anaconda/Nextflow pipeline in Ubuntu 16.04, 18.04, and in the recent 20.04 releases, respectively). Full description of how to install and run our pipeline is available on the annotate_my_genomes GitHub repository, please see: https://github.com/cfarkas/annotate_my_genomes#ii-installation

Reviewer #2:

The paper entitled "annotate_my_genomes: an easy-to-use pipeline to improve genome annotation and uncover neglected genes by hybrid RNA sequencing" describes a novel pipeline for enhancing existing genomic annotations using reference-based transcriptome assemblies, more specifically, obtained with 2nd and 3rd generation sequencing technologies combined. To demonstrate the pipeline usability, the authors apply their pipeline to the chicken genome and present a correct annotation of the extremely large SSPO gene, as well as a few other useful examples. Considering the rapid growth of interest in long RNA reads, this pipeline can become a valuable instrument for the scientific community and help researchers without a strong computational background.
The manuscript is fairly-written and easy to follow. The tool itself is available on GitHub, documented, easy to install and launch. Below I summarize a few issues, which, in my opinion, may improve the quality of the manuscript and the tool itself.

Major comments:

Q1. Although the authors present a new annotation pipeline, they do not cite or compare their tool with the existing approaches, such as for PASA [1] or tappAS package [2] (e.g. IsoAnnot-lite in particular: https://isoannot.tappas.org/isoannot-lite/), the latter of which is designed specifically for long-read analysis. I suggest the authors extend the Background section (and probably Discussions) by adding this comparison and providing information on how their pipeline is different from the similar existing instruments and what novelties they bring to the community.

Ans1. We thank the reviewer, and we agree with this suggestion. We benchmarked our method comparing the most similar pipelines AUGUSTUS, BRAKER1, BRAKER2, TSEBRA and SQANTI3 (from tappAS) using five different model organisms (Chiken (Gallus gallus), Mouse (Mus musculus), Human (Homo sapiens), Zebrafish (Danio Rerio), and the C.elegans worm). We didn't select the PASA pipeline as it only employs EST/Illumina RNA-seq reads. The results of this benchmarking are included along with the manuscript, Figure 3, and supplementary Figure 3, demonstrating that our method outperformed the referred pipelines when genome-guided StringTie assemblies are employed as inputs.

Q2. I downloaded the pipeline, it was fairly easy to install (not taking into account conda bugs) and to launch it. Unfortunately, I was not able to complete the run. Since the main outcome of this project is the novel computational pipeline, I feel like its usability is one of the most important aspects.
During the run I experienced a few problems:
a. First time I encountered this error message:
::
: 2. Writting novel discoveries to Stats.txt :

::
cat:
UCSC_compare./Bmo/prjbel/lrgasp/stringtie/mouse/ont_cdna/stringtie.gtf.t
map: No such file or directory

After moving stringtie.gtf into the folder, from which the pipeline was launched, the problem disappeared. However, a user should be able to provide an annotation from any location.

b. The second problem I encountered was this:

:::
: 5. Performing gene annotation by using GAWN pipeline :
:::
: Downloading GAWN annotation folder. See
https: /github.com/enormandeau/gawn.git
Cloning into 'gawn' .
remote: Enumerating objects: 342, done.
remote: Counting objects: 100% (51/51), done.
remote: Compressing objects: 100% (48/48), done.
remote: Total 342 (delta 30), reused 11 (delta 3), pack-reused 291
Receiving objects: 100% (342/342), 133.68 KiB | 1.17 MiB/s, done.
Resolving deltas: 100% (189/189), done.
Done
cp: cannot stat 'gawn_config.sh': No such file or directory

Currently, for me it looks like the pipeline lacks testing on third-party computers and with various scenarios. Also, I recommend (1) adding --output (-o) option for specifying the output folder and (2) making it possible to run the pipeline from any folder, not just the one where it is located.

Ans2. We completely agreed with the reviewer, and now, as mentioned earlier, we have tested our pipeline on different operating systems (it works for OS Ubuntu 16.04, 18.04, and 20.04 LTS, respectively). We also add the option to specify the output folder, making it possible to run the pipeline from any location on the computer. Any additional issue can be reported through the GitHub page (https://github.com/cfarkas/annotate_my_genomes/issues/3) as it has been addressed since the few months that our pipeline is public.

Minor comments:

Q3. The usage of the most tools in the analysis seems fair. However, I have a few concerns regarding the upstream analysis:

a. The authors run minimap2 for aligning short-read RNA-Seq data. However, to the best of my knowledge, minimap2 does not support spliced alignment of short reads. Minimap2 has a preset -x sr for short genomic reads and -x splice / -x splice:hq for RNA Nanopore / PacBio CCS reads respectively. I presume splice short reads might be missing from the alignment and suggest using STAR [3] or HiSat2 [4] for mapping short-read RNA-Seq data.

Ans3. We agreed with the reviewer, indeed the PacBio reads are aligned using Minimap2. In contrast, Illumina short reads are aligned using HISAT2.

Q4. The authors use StingTie for reference based assembly. Although this tool is surely one of the best, they have now released an updated version StringTie2 [5], which was developed specifically for long read assembly. I wonder which version of StingTie was used in the analysis? Although the results seem to be fair, including assembly of such a huge gene as SSPO, I feel like a more accurate upstream analysis could result in even better results.

Universidad de Concepción

Research Institute in Oncology and Hematology

Ans4. We agreed with the reviewer, indeed our pipeline uses StringTie2 as assembler.

Q5. The authors mention rnaQUAST as a tool for assembly QC. This tool is typically used for de novo transcriptome assemblies, but can be used for reference-based assemblies as well. I suggest to mention and cite SQANTI [6], which is developed specifically for quality evaluation of long-read reference-based transcriptome assemblies.

Ans5. We agreed with the reviewer and included SQANTI3 in our manuscript.

Q6. The Data Description section contains the description of the entire pipeline. To the best of my knowledge, this section is reserved for describing the datasets only. I suggest moving this brief description to the beginning of the Analyses section (or Methods).

Ans6. We thank the reviewer for this observation, we have modified the Data Description.

Q7. The authors keep some of the abbreviations undisclosed (e.g. HH23 and HH30).

Ans7. We thank the reviewer for this observation, we have disclosed the abbreviation on the manuscript.

Q8. I suggest the authors provide versions for all tools being used, as well as the exact parameters that were used (especially for tools used outside the pipeline).

Ans8. We agreed with the reviewer and specified the version of the tools used in the present work. The detailed list is available on our Github page (please see: https://github.com/cfarkas/annotate_my_genomes/blob/master/environment.yml).

Best regards,

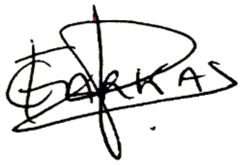

Corresponding authors: Dr. Carlos Farkas[1,2], Estefanía Tarifeño-Saldivia[4] and Teresa Caprile[3]
1: CancerCare Manitoba Research Institute, Winnipeg, MB, Canada.
2: Department of Pharmacology and Therapeutics, Rady Faculty of Health Sciences, University of Manitoba, Winnipeg, MB, Canada
3: Departamento de Biología Celular, Facultad de Ciencias Biológicas, Universidad de Concepción, Chile
4: Departamento de Bioquímica y Biología Molecular, facultad de Ciencias Biológicas, Universidad de Concepción, Chile
E-mail: carlos.farkaspool@umanitoba.ca, etarisal@udec.cl, tcaprile@udec.cl
