# Supplementary figures and images for: annotate_my_genomes: an easy-to-use pipeline to improve genome annotation and uncover neglected genes by hybrid RNA sequencing
Source: Gigascience. 2022 Dec 6;11:giac099. doi: 10.1093/gigascience/giac099 (PMC9724561; doi:10.1093/gigascience/giac099)

A

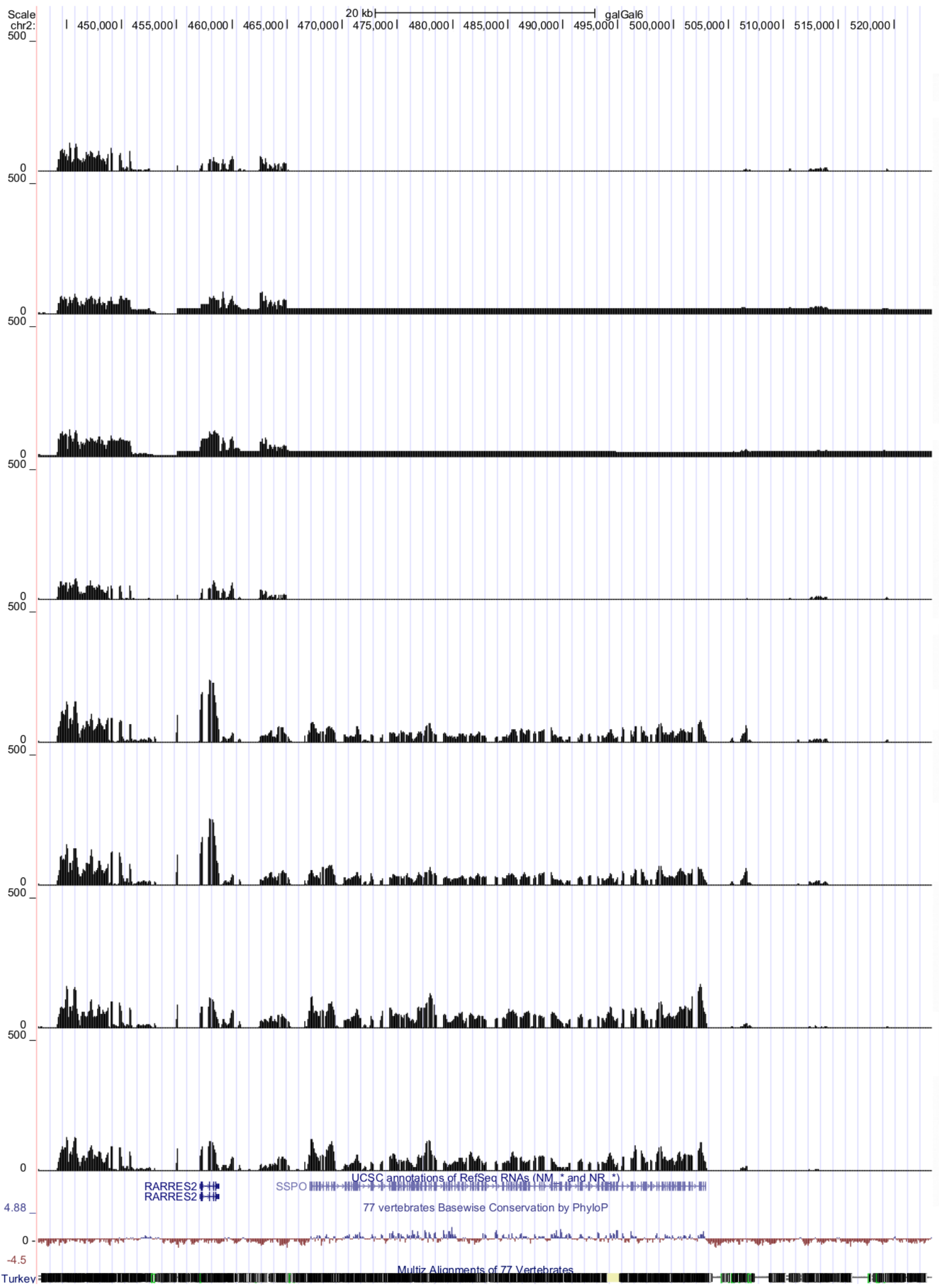

Supplement: giac099_Supplemental_Files [file giac099_supplemental_files.zip › Supplementary_Figure_1.pdf]

A

All Isoforms

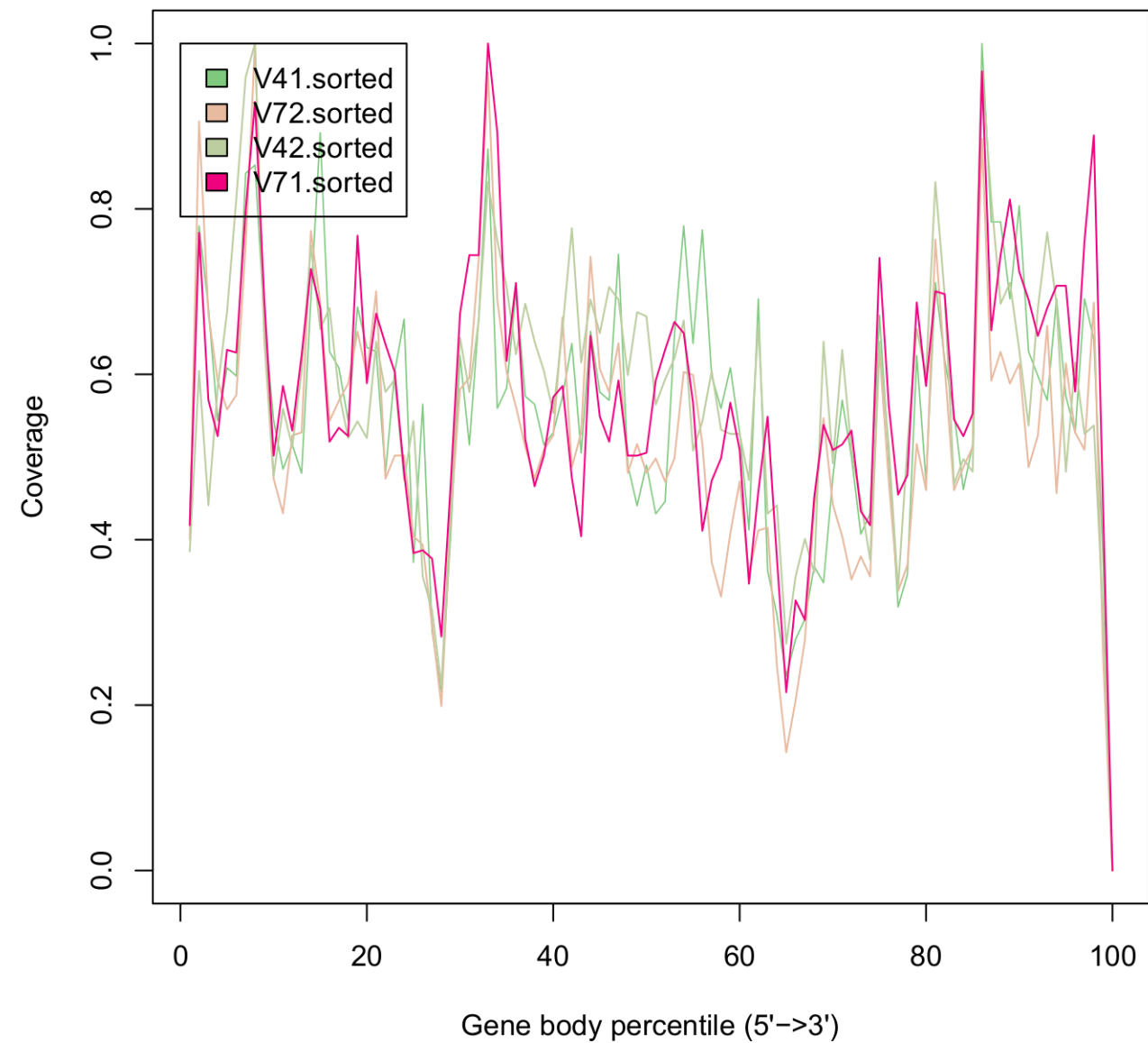

B

5' and 3' Isoforms

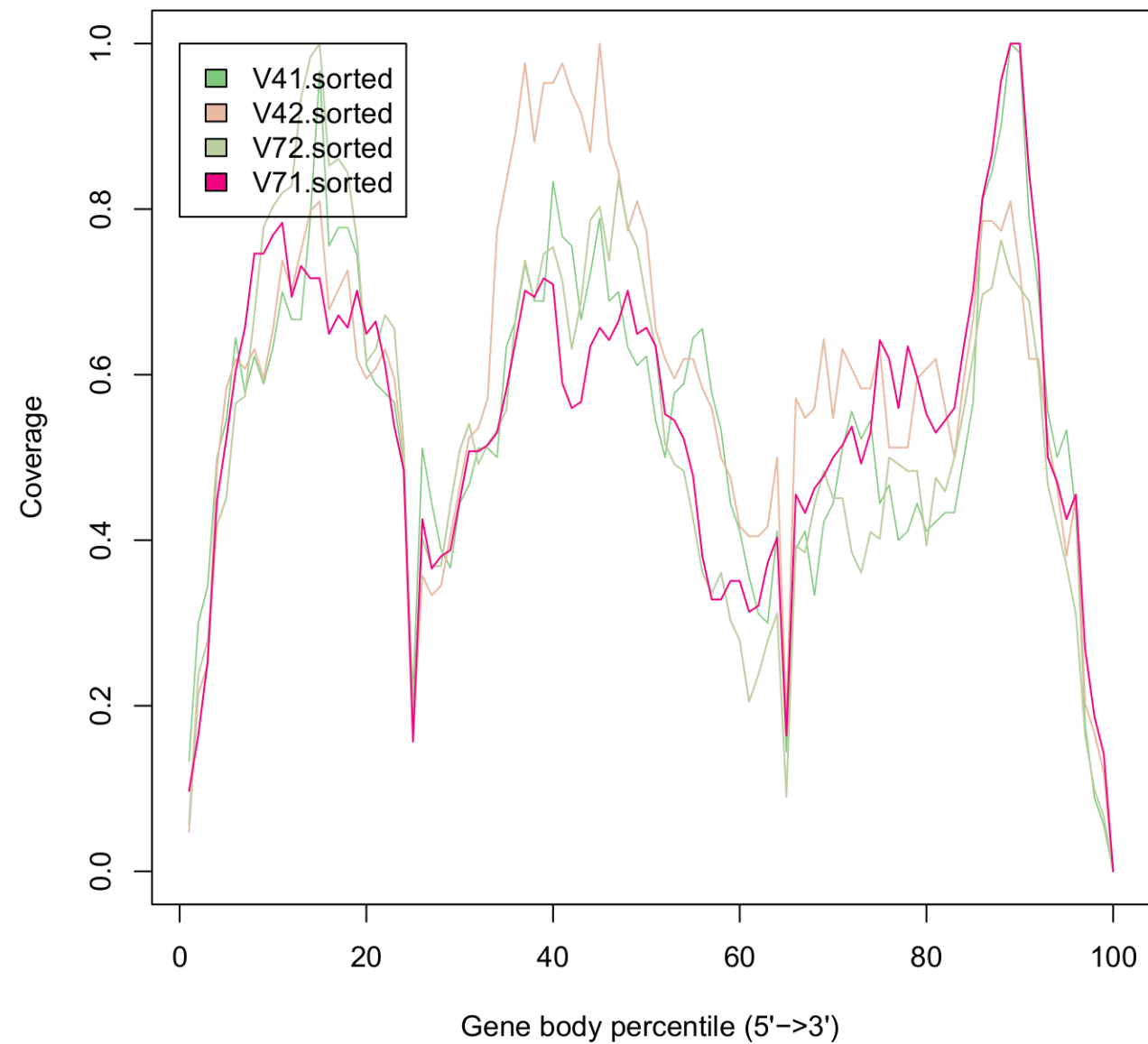

Supplement: giac099_Supplemental_Files [file giac099_supplemental_files.zip › Supplementary_Figure_2.pdf]

**A**

**galGal6**

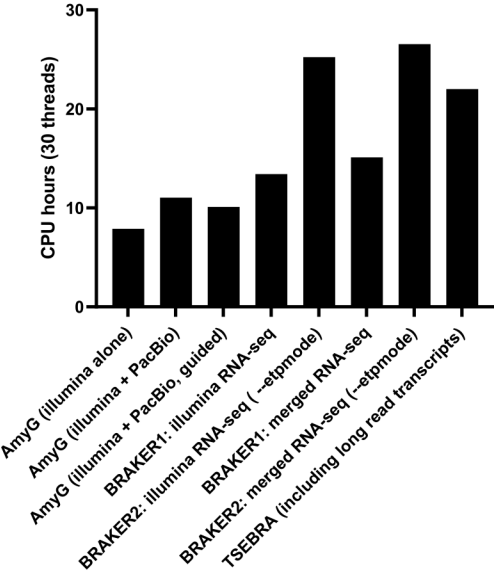

**B**

**mm10**

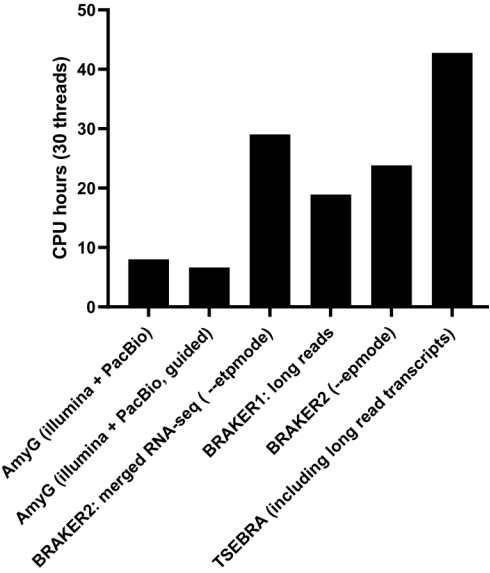

**C**

**hg38**

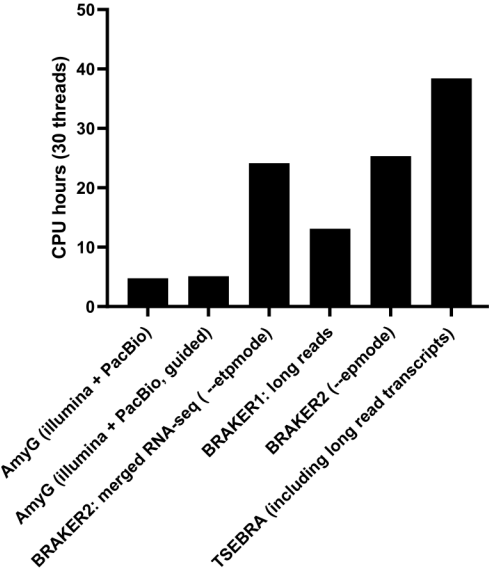

**D**

**danRer11**

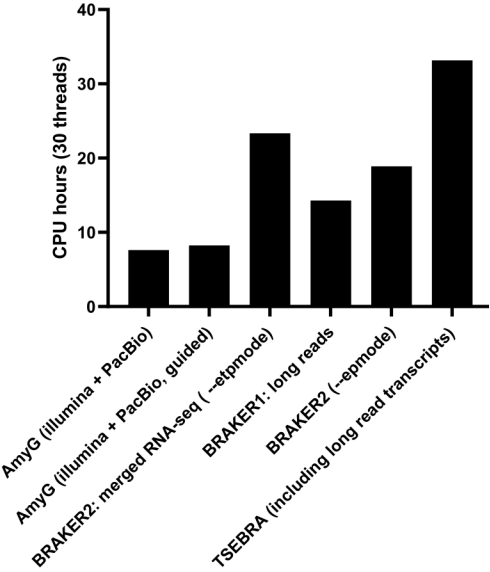

**E**

**ce11**

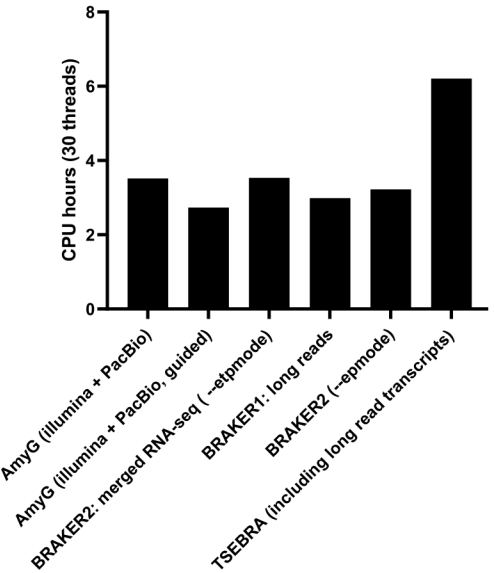

Supplement: giac099_Supplemental_Files [file giac099_supplemental_files.zip › Supplementary_Figure_3.pdf]
