# Supplementary material for: annotate_my_genomes: an easy-to-use pipeline to improve genome annotation and uncover neglected genes by hybrid RNA sequencing
Source: Gigascience. 2022 Dec 6;11:giac099. doi: 10.1093/gigascience/giac099 (PMC9724561; doi:10.1093/gigascience/giac099)
Supplement: giac099_Response_to_Reviewer_Comments_Original_Submission [file giac099_response_to_reviewer_comments_original_submission.pdf]

Author's Response To Reviewer Comments

Close

Dear Dr., Nogoy
We are pleased to resubmit our manuscript entitled “annotate_my_genomes: an easy-to-use pipeline to improve genome annotation and uncover neglected genes by hybrid RNA sequencing”. We have carefully responded to each reviewer’s concerns, and hope that it is now acceptable for publication in GigaScience.
In this new manuscript version, as suggested by reviewer #2, apart from our previous benchmarking with real next-generation sequencing datasets, we propose a more rigorous benchmarking including the use of simulated Illumina and PacBio datasets when comparing published genome-annotation methods against annotate_my_genomes. In this manner, we largely removed the bias of coverage and inherent errors from real next-generation sequencing datasets within species. Also, we reorganized the GitHub repository where annotate_my_genomes pipeline is hosted separating pipeline installation from running steps. Performance and innovative aspects of our pipeline are included in the discussion section. Because of the concerns of Reviewer 2 about the pipeline implementation, the new version of the manuscript includes alternative ways to implement the pipeline through containers. We re-wrote the entire pipeline into NextFlow native scripts, and we also implemented annotate_my_genomes via Docker. In this version, we explicitly recommend running annotate_my_genomes via NextFlow.
The manuscript now presents one additional file (Supp3-SSPO-annotation.xlsx) and one new Figure (Figure 4), therefore, old Figure 4 now becomes Figure 5. In this way, we present five main figures, three supplementary figures, and five supplementary tables. Below, is our point-by-point response to the reviewer’s comments:

Reviewer 1:

Reviewer #1: The manuscript "annotate_my_genomes: an easy-to-use pipeline to improve genome annotation and uncover neglected genes by hybrid RNA sequencing" by Farkas et. al. presents a pipeline to annotate genomes with available genome annotations. The manuscript is written concisely. The authors present a methodology to integrate long-read RNA-Seq data with short-read to generate the assembly. The annotation pipeline uses a combination of Illumina (short-reads) and PacBio (long-reads) sequencing which is reported by other studies that improve the quality of the assembly. The authors test the annotation pipeline with multiple organisms. They can identify and annotate genes that were missed in the chicken public reference genome. In this version, the authors added a Nextflow implementation of their pipeline that make their approach easy to use in multiple computational infrastructures. The authors have addressed all questions and comment I did in the previous revision. Therefore, I recommend this manuscript for publication.

R: We thank and acknowledge the assessments of this reviewer regarding our manuscript.

Reviewer 2:

General comments
The authors did a great job by addressing most of the comments and adding the benchmarks to the manuscript. However, despite substantial improvements compared to the previous version I still have a few concerns regarding the benchmarking and usability of the pipeline itself.

Major comments
1. Although the pipeline is wrapped in Nextflow, it seems like all the steps are launched by a single binary file (bin/annotate-my-genomes), which basically eliminates some of the Nextflow’s important features, such as the possibility to continue halted runs and file management. What is the main reasoning for not switching to Nextflow entirely?

R: We thank the reviewer for this suggestion. We now implemented the entire pipeline into NextFlow native language as NextFlow scripts and we encourage users to launch the pipeline using NextFlow over the use of binaries. Now, users can launch the pipeline on NextFlow as described here: https://github.com/cfarkas/annotate_my_genomes#iii-running-the-whole-pipeline-via-nextflow-recommended.

2. Benchmarking is a significant addition to the paper. I also appreciate that authors created a separate benchmarking wiki page on GitHub. However, I have a few questions.

a. The benchmark uses real sequencing data, which may contain unannotated isoforms (i.e. even human and mouse annotations are not perfect). Thus, even perfectly restored novel isoforms will be considered as false positives when using RefSeq annotation as a ground truth. I suggest adding a single example on simulated data where the ground truth is known precisely. For simulating long RNA reads I suggest using IsoSeqSim (github.com/yunhaowang/IsoSeqSim) or NanoSim (github.com/bcgsc/NanoSim). Moreover, I may provide some data if the authors would like to carry out this experiment, don't hesitate to contact me.

R: We also thank this reviewer for the valuable suggestion on the manuscript. We simulated both PacBio and Illumina reads for Gallus gallus, Mus musculus, Homo sapiens, Danio rerio and Caenorhabditis elegans species, respectively. For PacBio reads, we employed IsoSeqSim tool using as inputs the genome (FASTA format) and the NCBI reference GTF from each of the referred species. In this manner, we designed a set of curated transcripts from NCBI on each species as ground truth. As suggested on the IsoSeqSim GitHub repository, we simulated substitutions/mismatches (up to 1.731%), deletions (up to 1.090%), and insertions (up to 2.204%) based on the percentages observed by the Alzheimer's disease brain Iso-Seq data released by PacBio in 2016 (https://downloads.pacbcloud.com/public/dataset/Alzheimer2019_IsoSeq/). We established the average read count per transcript to 20 (--nbn parameter). The description of this methodology was integrated into the manuscript between lines 531-538 (red letters).
To simulate illumina data, we mapped the real illumina reads datasets employed in the benchmarking from each species against their reference transcriptome (in FASTA format) using bowtie2 aligner with the parameter -X equals to 2000. The sorted aligned BAM files along with the reference transcriptome (in FASTA format) from each species were used as input to simulate paired illumina datasets per species. Importantly, to fulfill fair comparisons between C. elegans dataset compared to the others, we included Illumina reads for this species since we previously evaluated nanopore-based direct RNA sequencing of Caenorhabditis elegans embryos without using illumina data. We simulated illumina reads using ReSeq illuminaPE mode using the parameters -j 50 and -c 20, respectively. The description of this methodology was integrated into the manuscript between lines 540-547 (red letters).
After these steps, PacBio and Illumina simulated datasets were aligned against their reference transcriptomes using minimap2 (using -ax splice flag) and HISAT2 aligners on default mode, respectively. The resulting simulated transcriptomes were merged and assembled using StringTie or inputed for StringTie-mix assembler. The resulting GTF files were inputted into annotate_my_genomes pipeline to obtain annotated GTF files while the individual Illumina and PacBio alignments in BAM format were used as inputs for BRAKER1/2 and TSEBRA pipelines, respectively. The precision, recall, and their harmonic mean (F1-score) were obtained by using gffcompare.
According to the F1 scores, the results of this benchmarking using simulated reads indicated that annotate_my_genomes using either de novo StringTie assemblies or merged PacBio+Illumina transcriptome assemblies (in genome-guided mode) is better than the competitor methods while reconstructing the transcriptome of each of the evaluated species. These results are summarized on Figure 3E and stated between lines 263-280 of the manuscript (red letters).
Moreover, the use of the novel assembly method StringTie mix (https://doi.org/10.1371/journal.pcbi.1009730, published during the revision of our manuscript) in genome-guided mode outperform all previously tested methods in all evaluated parameters. Therefore, to achieve the optimal results, we recommend using StringTie mix raw GTF annotations as input for our pipeline to increase genomic annotations with high confidence. These results are summarized on a new Figure 4E and stated between lines 281-321 of the manuscript (red letters). Legend of new Figure 4 was included between lines 664-689 of the manuscript.

b. The F1-scores for transcript level on mouse and human data look rather low for most tools, especially for

unguided mode. Low recall can be explained by the fact that some of the reference isoforms are not expressed, and thus could not be reconstructed (although gffcompare is used with -r, all overalping isoforms would still be used as a ground truth). However, what is the main cause of the low precision, false positive predictions reported by StringTie or false annotations? It could be useful to give F1-scores for the raw StringTie annotations as well.

R: We agree on this point with the reviewer as low recall can be explained by missing gene/isoform expressions from the nature of the real datasets itself. However, the recall at the locus level greatly improved on the real human data by using StringTie-mix assembly as input for our pipeline (Figure 3D vs 4D). In an ideal scenario, when all reference transcriptome is covered (simulated datasets) our pipeline greatly surpass precision and recall values from the other methods (Figure 3E and 4E, respectively). Therefore, we assume lower recall values are related to the real dataset coverages and not for the failure of our pipeline itself.
Regarding raw StringTie annotations versus the processed GTF from our pipeline, it was proven that our pipeline did not sacrifice F1-scores from these raw annotations. As a matter of fact, our pipeline improved de novo StringTie raw annotations at the intron chain, transcript, and locus fields (Figure 4D) and maintained F1-scores with StringTie genome-guided annotations, including StringTie mix raw annotations. Therefore, we maintained our conclusions regarding the benefits to implement our pipeline coupled with the StringTie assembler. These observations are described in the manuscript between lines 302-324 of the Manuscript.

c. While annotate_my_genomes shows an improvement for most of the datasets, unguided mode seems to have slightly worse results compared to the competitors on C.elegans dataset with respect to transcript and intron chain levels. Why could the results be different on this particular dataset?

R: Concerning this point, the used dataset corresponded to Larvae L1 cells from C. elegans sequenced by direct nanopore RNA-seq technology, not relying on Illumina short read technology. However, to assess the reviewer’s comment, we simulated Illumina RNA-seq reads for this specie and include the simulated reads in the analysis (Figure 3E and 4E, respectively). In C. elegans simulated data, both transcript and intron chain levels F1-scores from our pipeline were superior in comparison with all other methods.

Minor comments
1. I would suggest improving the user manual, e.g. add table of contents and subsections. For example, I was running the example provided in the manual, but since it is not launched via Nextflow, it requires all packages installed via Conda, which may not be clear for an inexperienced user. The installation section should contain only information about the installation, not on running the pipeline.

R: We updated the installation section, by removed the running steps from this section. Now, in our repository the installation section corresponds to section II and the running steps via NextFlow and Anaconda + Binaries are summarized in section III and IV, respectively. For reproducibility, we encourage to implement the pipeline via NextFlow rather than Anaconda.

2. Running “bash makefile.sh” yielded an error (but proceeds to the end eventually):

gzip: swissprot.tar.gz: invalid compressed data--format violated
tar: swissprot.tar: Cannot open: No such file or directory
tar: Error is not recoverable: exiting now
R: This error was related to the download of the swissprot database itself, due bad file size (corrupted file). Since the downloaded swissprot database was not correct in terms of the expected byte size, gzip raised the invalid compressed data error and therefore, tar was unable to decompress the entire database. We tested at least six different computers and we were able to reproduce the exact error in one of them. To correct this error and ensure reproducibility, the pipeline now can be installed directly from NextFlow by using makefile.nf file hosted in the GitHub repository. In this setting, the wget and gzip commands are run via NextFlow container, not relying on local wget and gzip versions when ‘bash makefile.sh’ is employed.

3. Running the pipeline requires creating the output folder beforehand — I believe the pipeline can create it automatically. However, all the output is written to another folder anyway

(annotate_my_genomes_DATE_TIME).
R: We thank the reviewer to raise this point. We recognize that at some point, it was confusing the output specification. To correct this weakness, we implement that the users must create an output folder first and the pipeline will output on it. But also, users can specify a non-empty given directory. If annotate_my_genomes find the user-specified output directory is not empty, it will automatically create the annotate_my_genomes_DATE_TIME output directory and it will output on it. This practice prevents the pipeline to output in a non-empty folder (that might contain many files) and in this way prevent output data loss.

4. I cannot say for sure whether the pipeline has finished correctly or not — I recommend implementing better error handling with clear messages as well as a message about successful finish. My both runs (example and my own mouse data) finished with:
::: FEELnc calculations were done. The output is called candidate_lncRNA_classes.txt :::
::
::: 8. Parsing GAWN and FEELnc outputs :::
::
Seems like the run was not entirely successful.

R: From the error raised from point 2 (that led to a malformed swissprot database), probably the run failed from the BLASTX step from GAWN annotation pipeline. With a failed swissprot database, the BLASTX step also failed, and the error propagated until step 8 when the pipeline stopped (as outputted in the shell). These errors can be properly documented by running the pipeline on NextFlow. Thus, we recommend users running annotate_my_genomes pipeline from NextFlow to have a proper log and full reproducibility of potential errors.

5. To the best of my knowledge, SQANTI3 is a transcript classification tool rather than annotation, thus it shouldn't be used in the benchmarking.
R: We partially agree with the reviewer on this point. Although SQANTI3 was designed for quality check of long read sequencing RNA-seq datasets (including hybrid RNA-seq datasets) the SQANTI3 pipeline can also provide an assembly of a given transcriptome in the GTF format. We tested SQANTI3 only with our Gallus gallus long read sequencing datasets and no gene model was resolved on SSPO locus (Figure 3A). Therefore, as suggested by this reviewer, we removed the SQANTI3 comparisons from the benchmarking.

6. When running the pipeline via Nextflow, it always gets frozen at the "conda install -c conda-forge -y sed" step. Isn't sed a basic Linux tool and installing it via conda necessary?

R: We acknowledge the reviewer for reporting this issue. As asked by this reviewer, we removed the sed install from the conda recipe. This updated conda recipe was already tested on NextFlow or as anaconda environment, hosted in both Ubuntu 18.04 and 20.04, respectively. To ensure maximal reproducibility, we also implemented the whole annotate_my_genomes pipeline on Docker. In this manner, users need to download and install the repository (e.g.: achived via NextFlow), download the docker image, and run it as described here: https://hub.docker.com/repository/docker/carlosfarkas/annotate_my_genomes.

Regarding all valuable recommendations from the two previous reviewers, and now with the inclusion of real and simulated sequencing data for benchmarking, including the improvement of the pipeline implementation into NextFlow/Docker containers, we feel this manuscript is ready for publication. Nevertheless, if any errors are raised during test runs of the pipeline, we encourage Reviewer #2 to actively submit any errors into the issue section of our GitHub repository: https://github.com/cfarkas/annotate_my_genomes/issues. In this manner, we can readily work on new issues and keep records for other users.
We are thankful for considering this revised manuscript for publication. We present original research that has not been previously published and is not under review elsewhere. We declare no conflict of interest.

Sincerely,

Corresponding authors:
Carlos Farkas PhD, Estefanía Tarifeño-Saldivia PhD, and Teresa Caprile PhD.

Close
